# Hospital Presenting Self-Harm and Risk of Fatal and Non-Fatal Repetition: Systematic Review and Meta-Analysis

**DOI:** 10.1371/journal.pone.0089944

**Published:** 2014-02-28

**Authors:** Robert Carroll, Chris Metcalfe, David Gunnell

**Affiliations:** School of Social and Community Medicine, University of Bristol, Bristol, United Kingdom; Copenhagen University Hospital Gentofte, Denmark

## Abstract

**Background:**

Non-fatal self-harm is one of the most frequent reasons for emergency hospital admission and the strongest risk factor for subsequent suicide. Repeat self-harm and suicide are key clinical outcomes of the hospital management of self-harm. We have undertaken a comprehensive review of the international literature on the incidence of fatal and non-fatal repeat self-harm and investigated factors influencing variation in these estimates as well as changes in the incidence of repeat self-harm and suicide over the last 30 years.

**Methods and Findings:**

Medline, EMBASE, PsycINFO, Google Scholar, article reference lists and personal paper collections of the authors were searched for studies describing rates of fatal and non-fatal self-harm amongst people who presented to health care services for deliberate self-harm. Heterogeneity in pooled estimates of repeat self-harm incidence was investigated using stratified meta-analysis and meta-regression. The search identified 177 relevant papers. The risk of suicide in the 12 months after an index attempt was 1.6% (CI 1.2–2.4) and 3.9% (CI 3.2–4.8) after 5 years. The estimated 1 year rate of non-fatal repeat self-harm was 16.3% (CI 15.1–17.7). This proportion was considerably lower in Asian countries (10.0%, CI 7.3–13.6%) and varies between studies identifying repeat episodes using hospital admission data (13.7%, CI 12.3–15.3) and studies using patient report (21.9%, CI 14.3–32.2). There was no evidence that the incidence of repeat self-harm was lower in more recent (post 2000) studies compared to those from the 1980s and 1990s.

**Conclusions:**

One in 25 patients presenting to hospital for self-harm will kill themselves in the next 5 years. The incidence of repeat self-harm and suicide in this population has not changed in over 10 years. Different methods of identifying repeat episodes of self-harm produce varying estimates of incidence and this heterogeneity should be considered when evaluating interventions aimed at reducing non-fatal repeat self-harm.

## Introduction

Approximately half of all people who die by suicide have previously self-harmed [1] and within the UK, 15–20% visit a hospital for self-harm in the year prior to their death [2]. The rate of suicide in the self-harm patient population is up to 100 times higher than that of the general population [3]. Therefore self-harm presentations to hospital represent an important opportunity for suicide prevention.

A key indicator of the effectiveness of hospital management of such patients is the incidence of repeat self-harm, both fatal and non-fatal, following discharge from hospital. Non-fatal repeat self-harm is a negative outcome for both the patient and in terms of health care cost [4]. Repeat self-harm has also been associated with a further increased risk of suicide. Patients who present to hospital for self-harm more than once have approximately double the risk of subsequent suicide compared to those presenting only once [5].

In a systematic review published in 2002 the risk of non-fatal repetition in the year after an initial episode of self-harm was previously estimated to occur in 16% of hospital presenting cases [6]. Since this review the literature on the risk of repeat self-harm has expanded greatly including studies based on ever larger cohorts with many years of follow-up. Furthermore, there has been a growing body of research from Asia [7]–[10], as well as other continents. Some of the data from Asia suggest the incidence of repeat self-harm may be lower than in other parts of the world [11]–[13]. Investigating variation in the incidence of repeat self-harm internationally could lead to important insights into the optimal configuration of health care services.

Furthermore, there is growing recognition that estimates of repeat self-harm are influenced by the method of recording the repeat event. Different methods of recording repeat self-harm include specialist research databases, national hospital admissions data, as well as patient report. In a recent trial both patient reported and hospital attendance data were used to identify repeat self-harm [14]. In the control arm of the trail, hospital records indicated that 11% of patients had a repeat self-harm episode within a year after an initial presentation, this compared to an 18% repeat self-harm rate based on patient report. The impact of these different methods of data collection and their influence on estimates of repeat self-harm has not previously been assessed systematically.

This systematic review and meta-analysis aims to update previous estimates of the incidence of fatal and non-fatal repeat self-harm [6]. Furthermore, we investigate study characteristics and cohort characteristics and their impact on these estimates by using sensitivity analysis based on stratified meta-analysis and univariable meta-regression.

## Methods

### Search criteria

Medline, Embase and PsychInfo databases were searched (see search strategy in Appendix S1) using OvidSP to identify all papers published between 2000 and 2012 with no restrictions on the language of publication. A medical librarian was not involved in the development of the strategy. Papers identified in an earlier review (1970–1999, n = 90) were combined with those returned by our search. The current analysis therefore included papers published from 1970–2012. Citation searches of key papers [3], [6], [15] were undertaken using Google Scholar. The reference sections of included papers and personal paper collections of the authors' were also searched.

### Inclusion and exclusion criteria

The search was designed to identify papers describing people who presented to health care services (primary or acute care) for deliberate self-harm, with or without suicidal intent, that reported the rate of subsequent fatal or non-fatal repeat self-harm. We restricted the review to cohort studies and randomised controlled trials (RCTs). In the case of RCTs, information was only recorded from the control arm of the trial. Papers that included a cohort or control arm consisting of less than 50 participants, prior to any loss to follow-up, were excluded. Papers focusing on patients with specific disorders, such as schizophrenia or depression, or patients within a specific age group, were also excluded.

Where a single paper reported on data from different centres or time periods, each centre/time period was treated as an individual record. In some instances, multiple papers were published regarding the same cohort of patients with the same outcome. When duplicate publications were found, the papers that reported the most in-depth description of the characteristics of the cohort were selected for inclusion.

### Data extraction

All three authors reviewed the papers, with data extraction of each paper being undertaken by at least two of the authors to confirm continuity in data collection practices. Any discrepancies between the data extraction were discussed and agreed by consensus.

### Statistical analysis

The incidence of self-harm was recorded as the number of people who repeated after an index attempt in a certain time frame e.g. one year. In some studies the duration of follow-up varied depending on the time a patient was entered into the study. Where patients were recruited and followed-up over different periods of time (e.g. at any point between Jan 2000 and Dec 2002) a constant rate of recruitment was assumed and average follow-up calculated from the mid-point of recruitment to the end of follow-up. Follow-up time (whether equal for all patients or varied) and its association with estimated risk of repetition was investigated.

Estimates of repetition could also vary depending on the method used to calculate incidence. Some studies reported a crude number of patients repeating within a mean follow-up time while others used rate-based approaches or survival analysis which removed individuals from the risk set once they had experienced an event. In some instances, the raw data on number of repeat events was not reported; where possible the estimates were taken from survival plots. The impact of these different methodologies on estimates of repetition was investigated.

As well as recording the outcome, information on cohort and study characteristics that could influence repetition were recorded. Cohort characteristics studied included cohort gender and age, methods of self-harm (% self-poisoning) and previous self-harm. Study characteristics included year of publication, study design (RCT vs. Cohort study), outcome ascertainment (i.e patient report, A&E attendance, hospital admission), and continent (i.e. Europe, Asia, Americas). Outcome ascertainment for non-fatal repetition included repeat hospital admission, hospital attendance or patient report. All three methods of outcome ascertainment could be used by a single study. For the purposes of the analysis, studies were categorised according to the most comprehensive method they used, with admissions data considered as the least comprehensive and patient report being most comprehensive.

Pooled estimates of repetition were calculated using random effects meta-analysis. Meta-analyses estimated the incidence of non-fatal repeat self-harm at 1, 2 and 5 years and the same periods applied to fatal repetition, as well as 10 years. Between study heterogeneity was assessed using *Tau* ^2^ which has been suggested to be a more appropriate measure than *I* ^2^ when both the number of studies included and their respective sample sizes are large [16]. The proportion of patients repeating was recorded for each study. A numerator of 0.5 was used for studies reporting 0 repeat events during follow-up. Estimates of repetition were converted to logits for the meta-analyses, as these are unbounded and so allow easier calculation of confidence intervals [17].

The associations of study characteristics with estimates of repetition were investigated using meta-analysis stratified by the relevant study characteristic. Univariable meta-regression was used to obtain p-values and assess the statistical evidence of individual study characteristic's association with estimates of repetition. Statistical analyses were performed using Stata version 12.1 (Stata Corp, College Station TX, 2011).

## Results

**Figure 1:**
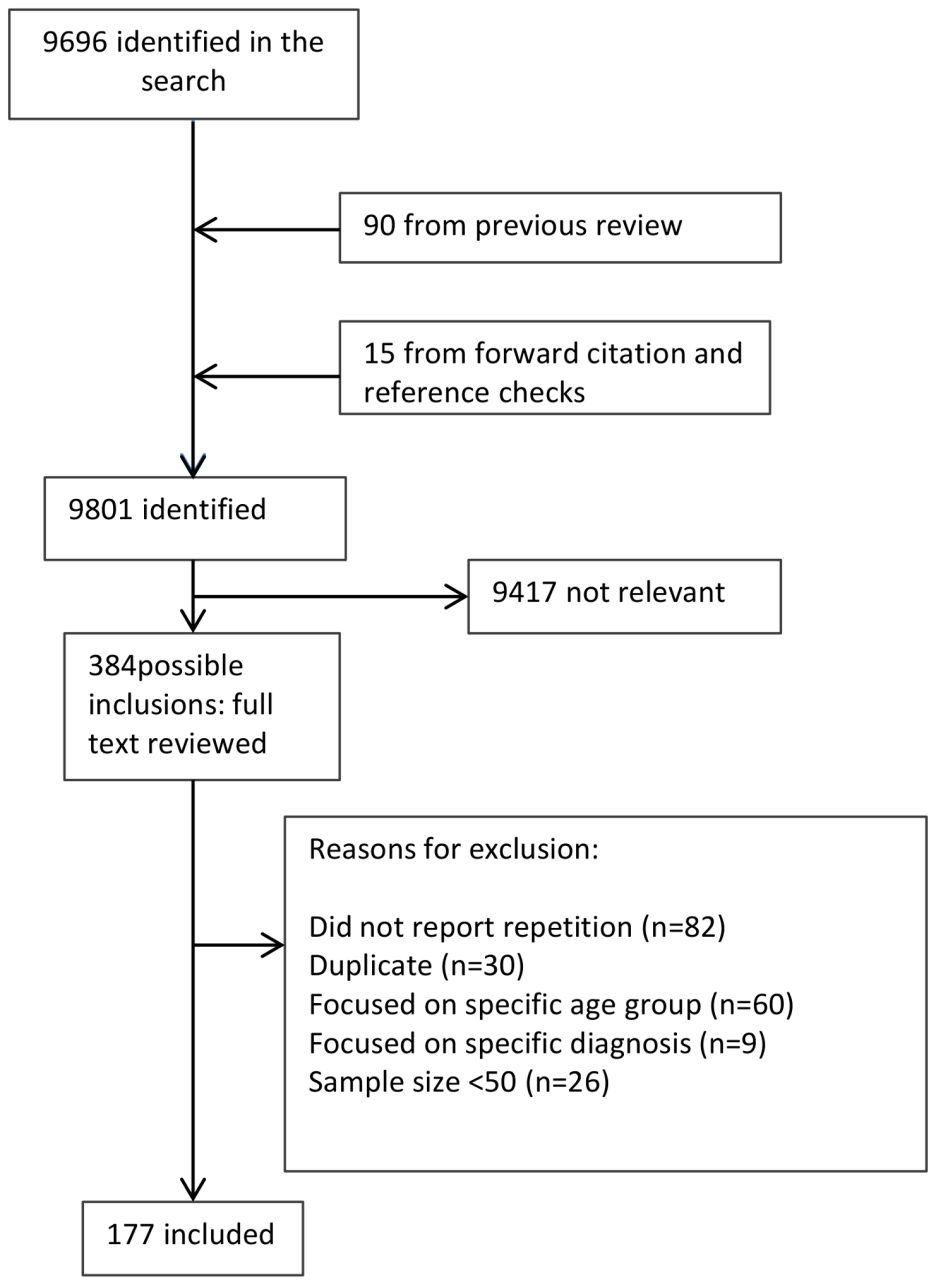
Literature review flow chart.

A total of 9801 papers were identified (Figure 1). The title and abstract of each paper was reviewed and the reason for exclusions noted. Of 384 papers highlighted as possibly relevant that had the full text reviewed, 82 did not report on the outcome of interest, 9 focused on a specific psychiatric disorder, 26 had a sample size less than 50, and 60 focused on a specific age group; these papers were therefore excluded. A total of 177 papers were identified as being eligible for inclusion (see Appendix S2).

### Studies included

Some studies reported an estimate of repeat self-harm at numerous points in time, for instance 1, 2 and 5 year repeat rates for one cohort, or reported on two or more separate cohorts in one paper. There were over 600 different estimates of repeat self-harm reported in the 177 papers. Almost half of the papers included either came from the UK (28.8%, 51/177), Sweden (10.2%, 18/177), or Norway (7.3%, 13/177). Altogether 78.5% (139/177) were from Europe, 7.9% (14/177) were from Australia and New Zealand, 8.5% (15/177) were from Asia, and 5.1% (9/177) were from North and South America. There were no studies identified from Africa.

The median sample size of studies included was 394 individuals (range 50-50891) with a median follow-up time of 2 years (range 0.1–37.0 yrs). The included papers were predominantly cohort studies (87.5%, 155/177) and the remainder were based on control arms of RCTs (13.0%, 23/177). Cohorts on average consisted of 40% males (range 17.5–78.0%) and the median age of patients was 34 (range 10–99).

Self-poisoning was most often reported as the method of self-harm used. In studies that reported the proportion of patients using this method, the median was 90% (range 46.8–100.0%). The median proportion of patients presenting with self-injury (cutting) was 10.5% (range 0.0–27.2%) and for other methods of self-injury (e.g. hanging, jumping, burning and others) it was 6.2% (range 0.0–100.0%).

### Incidence of non-fatal repeat self-harm

**Figure 2:**
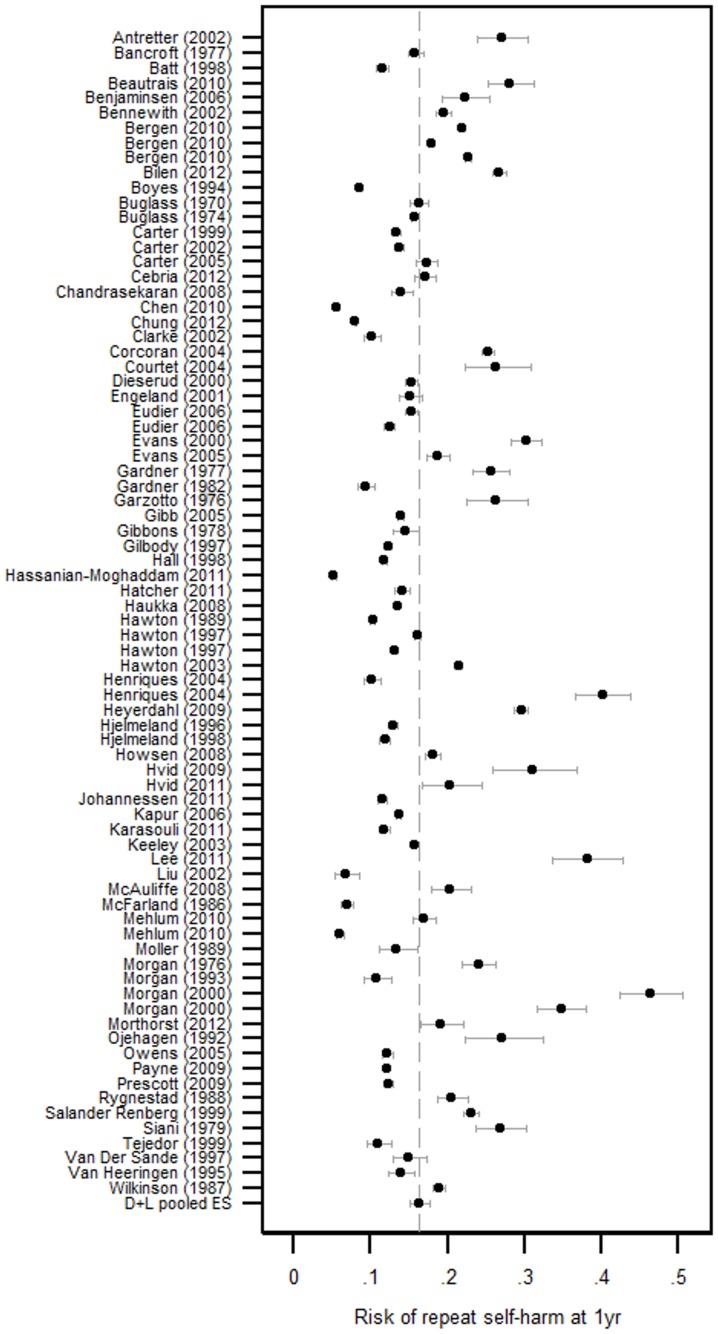
Forest plot of 1 year non-fatal repetition rates (%). Where findings were reported for several different centres/time periods/cohorts within one publication, results from each centre/time period/cohort appear separately.

**Figure 3:**
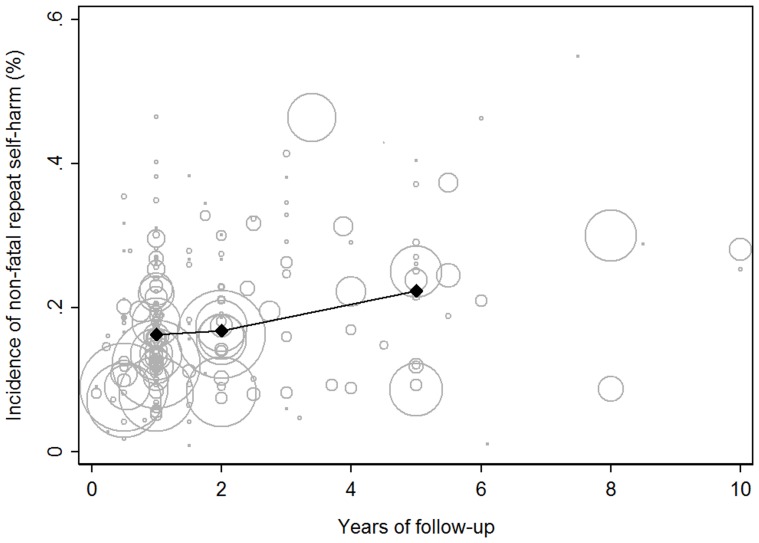
Individual study estimates of non-fatal repeat self-harm by duration of follow-up weighted by cohort size with overall pooled 1, 2 and 5 year estimates highlighted. Studies with follow-up over 10 years (n = 3) are not visible in this graph. Larger studies are indicated by larger circles.

The pooled estimated incidence of repeat non-fatal self-harm was 16.3% (95% CI 15.1–17.7) at 1 year (Table 1, Figure 2), 16.8% (95% CI 14.7–19.2) at 2 years and 22.4% (95% CI 17.0–28.9) at 5 years (Figure 3). Focusing on 1 year repetition, Tau^2^ = 0.1798 and a heterogeneity chi-squared test (χ^2^ = 34543, df = 79, p<0.001) suggested there was evidence of heterogeneity between studies greater than that expected by chance (*I* ^2^>90%). This heterogeneity between studies was investigated by patient characteristics, country/region of the study, and the method of reporting used to record the outcome. There was some evidence to suggest the proportion of patients with a history of previous self-harm in a cohort was positively associated with risk of non-fatal repeat self-harm within 1 year. Those cohorts with an above median proportion of patients previously self-harming (>43.7%) had an estimated 1 year repetition rate of 19.6% (95% CI 17.3–22.2), compared to 15.2% (95% CI 13.2–17.5) in those cohorts below the median (p = 0.021, Table 1).

**Table 1:** Meta-analysis estimates of the 1-year incidence of fatal and non-fatal repeat self-harm by cohort and study characteristics.

	% Non-fatal repetition				% Fatal repetition			
	1 year	95 CI	p	no. of studies	1 year	95 CI	p	no. of studies
Estimated Incidence	16.3	15.1–17.7	-	78	1.6	1.2–2.1	-	40
**Gender**								
Male	16.9	13.6–20.8	0.815	23	2.7	1.8–4.0	0.001	14
Female	16.4	13.1–20.4		23	1.2	0.7–1.9		14
**Age**								
Above median	17.9	14.2–22.2	0.533	17	2.4	1.9–2.9	<0.001	15
Below median	16.5	14.7–18.5		32	1.1	0.7–1.5		10
**Year published**								
> = 2000	17.2	15.5–19.1	0.800	49	1.4	0.9–2.2	0.492	17
<2000	14.9	13.5–16.3		29	1.7	1.4–2.1		23
**Proportion self-poisoning**								
Above median	15.3	13.8–16.9	0.926	28	1.1	0.9–1.4	0.020	12
Below median	16.9	14.4–19.8		29	2.0	1.2–3.2		15
**Previous self-harm**								
Above median	19.6	17.3–22.2	0.021	26	1.9	1.1–3.1	0.943	11
Below median	15.2	13.2–17.5		23	1.7	1.1–2.5		10
**Study design**								
RCT	15.5	11.6–20.3	0.609	15	1.0	0.5–2.0	0.055	7
Cohort	16.5	15.1–18.0		63	1.7	1.3–2.3		33
**Inclusion criteria**								
ED attendance	17.3	15.5–19.2	0.325	38	1.4	1.1–1.8	0.390	18
Hospital admission	15.5	14.1–17.0		41	1.6	1.3–2.1		19
**Outcome measure**								
Repeat attendance	17.0	15.2–18.8	0.011	38	-	-	-	-
Repeat admission	13.7	12.3–15.3		24	-	-		-
Patient Report	21.9	14.3–32.2		13	-	-		-
**Continent**								
Europe	17.1	15.9–18.4	0.076	62	1.6	1.2–2.2	0.986	33
Asia	10.0	7.3–13.6		6	1.7	0.9–3.0		3
New Zealand & Australia	16.3	14.5–18.4		7	1.4	1.1–1.7		3
North & South America	15.1	4.9–38.0		3	1.8	1.5–2.0		1

Meta-analysis stratified by method of follow-up suggested the rate of non-fatal repeat self-harm was 17.0% (95% CI 15.2–18.7) in studies based on repeat hospital attendances, 13.7% (95% CI 12.3–15.3) in studies based on repeat hospital admissions (admission to a hospital bed) and 21.9% (95% CI 14.3–32.2) in those based on repeat self-harm reported by patients. Univariable meta-regression revealed evidence (p = 0.011) that the method used to record repetition explained 9.3% of the between study variability in estimated 1 yr non-fatal repeat self-harm (Table 1).

By continent, the 1 year non-fatal repetition rate in European studies was estimated as 17.1% (95% CI 15.9–18.4) while there was weak evidence the rate was considerably lower in Asia (10.0%, 95% CI 7.3–13.6, p = 0.075; Table 1). The statistical evidence for this difference was strengthened when continent was dichotomised to Asian vs non-Asian estimates of repetition (p = 0.009). Univariable meta-regression suggested study location explained 5.3% of the between study variance in estimates. In a multivariable meta-regression, study location and follow-up type combined explained nearly a quarter (22.7%) of between study variation in estimated repetition rates.

In a stratified meta-analysis, there was no evidence (p = 0.118) to suggest the rate of repeat self-harm differed between studies based on follow-up time periods that were equal for all patients (17.1%, 95%CI 15.8–18.4) and studies based on cohorts that included patients with uneven follow-up time (14.0%, 95%CI 13.6–14.4). When comparing studies publish before and after the year 2000, there was no evidence to suggest the incidence of repeat self-harm had changed (Table 1) and this remained after controlling for the country the study was undertaken in and method of follow-up. Furthermore, while survival analysis is the most appropriate methodology for analysis of time to event data, there was weak evidence (p = 0.075) to suggest estimates of repetition differed between the studies that used survival analysis (13.9%, 95%CI 11.4–16.8) compared to studies that did not (17.3%, 95%CI 15.9–18.7).

### Incidence of fatal repeat self-harm

**Figure 4:**
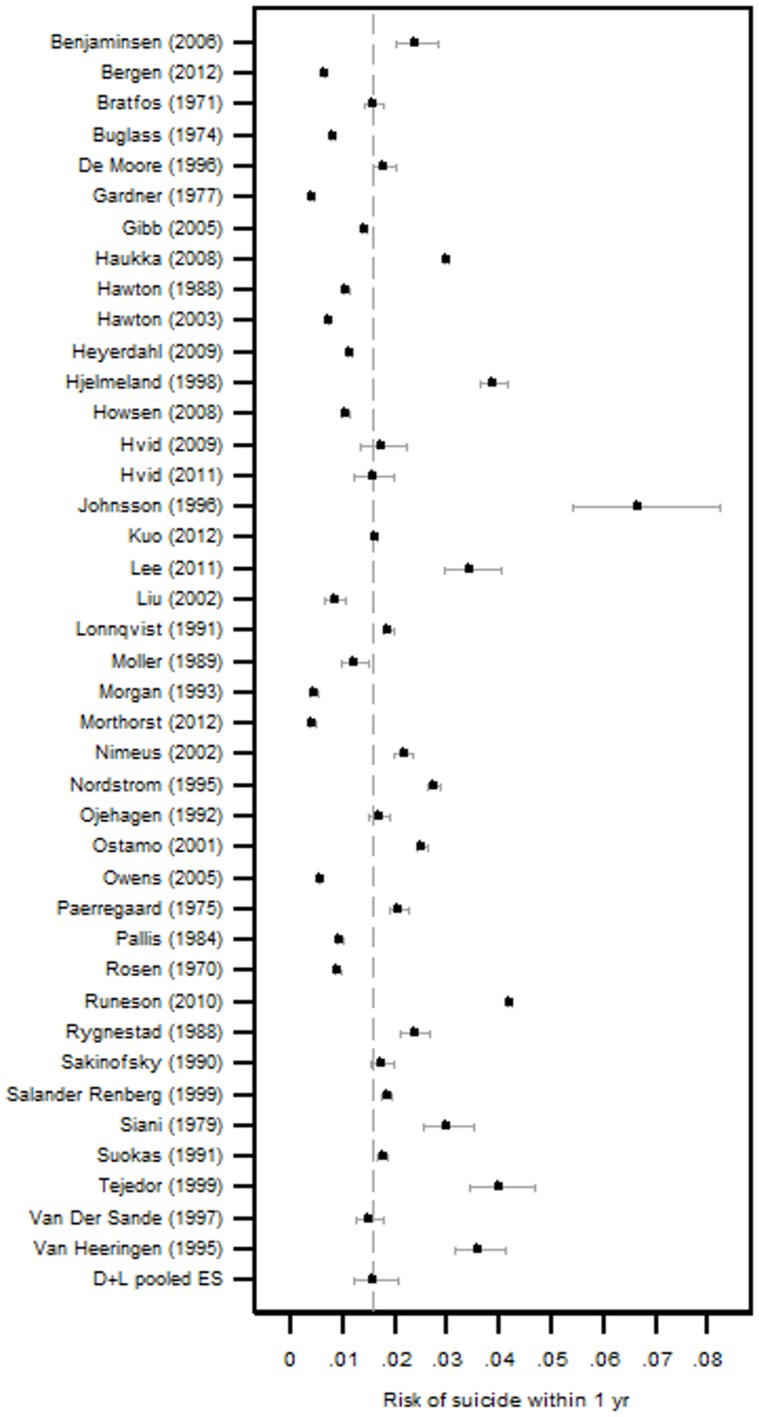
Forest plot of 1 year fatal repetition rates. Where findings were reported for several different centres/time periods/cohorts within one publication, results from each centre/time period/cohort appear separately.

**Figure 5:**
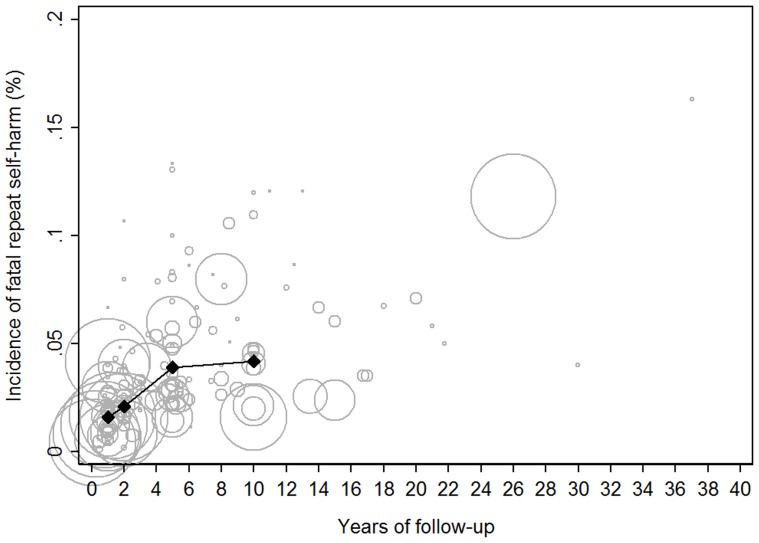
Individual study estimates of fatal repeat self-harm by years of follow-up weighted by cohort size with overall 1, 2, 5 and 10 year estimates. Larger studies are indicated by larger circles.

The pooled estimated incidence rate of subsequent fatal self-harm was 1.6% (95% CI 1.2–2.1) at 1 year (Table 1, Figure 4), 2.1% (95% CI 1.6–2.8) at 2 years, 3.9% (95% CI 3.2–4.8) at 5 years and 4.2% (95% CI 3.1–5.6%) at 10 years (Figure 5).

One year fatal repetition rates appeared to differ between males and females with a male estimate of 2.7% (95% CI 1.8–4.0) compared to 1.2% (95% CI 0.7–1.9%) in females (Table 1, p = 0.001).

Estimates of fatal repetition of self-harm were greater in cohorts where participants mean age was higher than average (Table 1, p<0.001). Those cohorts with average age above the median had an estimated 1 year repetition rate of 2.4% (95% CI 1.9–2.9) compared to 1.1% (95% CI 0.7–1.5) in those cohorts below the median. Age explained 40.1% of between study variation in estimates of fatal repetition and when sex was included, 69.4% of between study variation was explained. There was also some evidence that the proportion of a cohort using self-poisoning as a method at their index episode was inversely associated with risk of suicide. In cohorts with greater (above the median) proportions of patients self-poisoning, the 1 year fatal repetition rate was 1.1% (95%CI 0.9–1.4%) compared to 2.0% (95%CI 1.2–3.2) in those cohorts with less self-poisoning (Table 1, p = 0.035). Whether a study was an RCT or cohort study also appeared to have an impact on estimates of subsequent suicide, however controlling for cohort characteristics, including cohort age, weakened the evidence of this association. Other study characteristics, including year of publication, inclusion criteria, study location, and previous self-harm, did not appear to explain any of the between study variation in fatal repeat self-harm (Table 1).

## Discussion

Over 170 publications have investigated the incidence of repeat self-harm and suicide following hospital presentation for self-harm. The number of studies included in this meta-analysis is almost double that of the previous review in this area [6]. Estimates of repetition vary considerably. The key factors influencing these estimates are the country the study is undertaken in, the method used to collect the outcome data and the incidence of previous self-harm within the cohort. Studies based in Asian countries reported lower rates compared to estimates based on studies from different geographic regions. The overall 1 year rate of repeat self-harm was estimated as 16.3% but this varied from 13.7% when studies were based on hospital admissions data to 21.9% when data were based on patient report. Age, gender and method of self-harm explained a large proportion of the between study variation in estimates of suicide following self-harm. Fatal repeat self-harm was estimated to occur in 1.6% of people within 1 year after their index attempt and incidence was almost doubled in males compared to females (2.7% vs. 1.2%). This incidence of repeat self-harm and suicide is no lower in more recent published cohorts than in studies conducted over 14 years ago.

### Strengths and limitations

This meta-analysis updates the previous review in this area undertaken over a decade ago, drawing on a broad international literature. The review systematically investigated the impact of cohort level characteristics on estimates of the incidence of repeat self-harm and suicide. The number of studies in this review was large, adding power to subsequent analysis. Meta-regression can be prone to type I error when analyses are based on a small number of studies [18], the large dataset included in this study avoided the risk of such bias.

However, meta-analysis of observational studies comes with challenges inherent in the combination of a large number of studies with differing characteristics. There was a large amount of between study variation in estimates of repeat self-harm, and while up to 69.4% of this was explained, a large amount of heterogeneity remained unexplained. Heterogeneity in itself should not deter one from a meta-analysis [19]. While there were high levels of heterogeneity in estimates and study characteristics did vary, all cohorts were focused on hospital presenting self-harm and the incidence of subsequent repetition.

The ability to investigate heterogeneity within the meta-analysis was dependent on the reporting of study characteristics. A large number of studies included in this analysis did not report key information regarding the study cohort, reducing the power of our analysis and making it difficult to identify sources of heterogeneity between study estimates. None the less, study characteristics that could be ascertained from all cohorts gave important insight into how estimates of repeat self-harm differ, for instance, by country. A final limitation is that some included studies only reported repeat self-harm based on data from one centre; therefore repeat attendances made at other centres within the study catchment area may have been missed.

### Relation to other studies

The overall incidence of 1 year repetition in self-harm patients (16.3%) is in keeping with previous estimates [6]. The considerable variation in levels of repetition across studies appears to be related in part to the method of collecting information on repeat presentations. One of the most common and easily accessible sources of information on hospital presenting self-harm is national hospital admissions data (e.g. Inpatient Hospital Episode Statistics (HES) data in the UK). However, such data currently do not capture information on patients who present to the emergency department but are discharged without being admitted to a hospital bed. Admitted patients have been estimated to comprise approximately half of the hospital presenting self-harm population [20], however this proportion has been shown to vary considerably between institutions [21]. Studies that relied on admissions data therefore produced lower estimates of repeat self-harm than studies using other measures such as patient report.

The country a study was undertaken in also impacted on the incidence of repetition. Studies undertaken in Asia had considerably lower incidence of repeat non-fatal self-harm at 1 year compared to European studies (10.0% vs. 17.1%). The use of different measures of repeat self-harm did not explain the lower observed rate. The marked differences between rates of repetition may be due to patient characteristics as well as variations in hospital management. Pesticide self-poisoning in Asia is common and the case fatality is greater than 10% in some studies [22], [23]. This high case fatality may lead to fewer patients at risk of repetition [24]. Furthermore, the length of stay of patients admitted to a hospital in some Asian countries is greater compared to Europe, due to the high toxicity of methods commonly employed [24]. Risk of repetition is greatest immediately after an index episode [25] and this prolonged hospital stay may inhibit further suicidal behaviour during this high risk period.

As well as country and method of outcome ascertainment, previous self-harm was identified as a characteristic of study cohorts that explained variation in non-fatal repeat self-harm. Levels of previous self-harm within cohorts varied from 0% to 100% across studies. Previous self-harm has a well established association with risk of subsequent self-harm and it was identified as an important factor in the current analysis.

The incidence of repeat fatal self-harm in one year was 1.6%, increasing to 3.9% (95% CI 3.2–4.8) by 5 years and 4.2% (95% CI 3.1–5.7%) at 10 years. The few studies with follow-up extending beyond these time periods suggested risk continued to increase (Figure 5). These findings suggest risk of suicide persists well after the index self-harm episode. Between study heterogeneity in estimates of fatal repeat self-harm was explained in large part by two well documented risk factors for suicide; age and gender. Males have been consistently shown to be at elevated risk of suicide compared to females. Age was also identified as explaining between study heterogeneity with the average age of a cohort being positively associated to estimates of fatal repetition. Older patients presenting for self-harm have been previously identified as a high risk group for subsequent suicide [3].

Self-poisoning is the most common method used in hospital presenting self-harm and patients using this method are more likely to receive specialist psychosocial assessment and aftercare than those using self-injury [26]. However, patients presenting for self-harm, that use self-injury as a method, may represent a higher risk population for fatal repetition. A number of cohort studies have estimated greater risk of subsequent suicide in people who present initially with self-injury compared to those self-poisoning [27], [28]. This association was confirmed across studies included in the current analysis, with risk of suicide doubled in cohorts where the proportion of patients self-poisoning was below the median compared to those cohorts above the median.

### Conclusions

We estimate 1 of every 25 self-harm patients will go on to die by suicide in the 10 years after their index presentation. This risk is greater in older patients, males and those using methods other than self-poisoning. Suicide prevention efforts might usefully focus on these high risk groups. Estimates of the incidence of non-fatal repeat self-harm vary according to the country the study is undertaken and by the method used to collect data on repetition. The overall rate of non-fatal repetition of self-harm within 1 yr was 16.3%. Estimates of repetition based on hospital admissions data were lower, as not all patients require admission to a hospital bed. A higher rate of repetition was estimated from studies using patient report. This likely reflects the greater burden of disease within the community and is an important measure from a public health perspective. Future studies aimed at reducing non-fatal repetition of self-harm need to consider the best measure of repeat self-harm (self-report/hospital attendance/hospital admission) in order to accurately evaluate the impact of any intervention. Despite over 30 years of research in the area, the incidence of non-fatal repeat self-harm has not changed and this highlights the need for new approaches.

## Supporting Information

Appendix S1 **Search strategy.**
(DOCX)

Appendix S2 **Characteristic of included studies.**
(DOCX)

Checklist S1 **PRISMA statement.**
(DOC)

## References

[1] Foster T, Gillespie K, McClelland R, Patterson C (1999) Risk factors for suicide independent of DSM-III-R Axis I disorder. Case-control psychological autopsy study in Northern Ireland. Br J Psychiatry 175: 175–179. 10627802 10.1192/bjp.175.2.175

[2] Gairin I, House A, Owens D (2003) Attendance at the accident and emergency department in the year before suicide: retrospective study. Br J Psychiatry 183: 28–33. 12835240 10.1192/bjp.183.1.28

[3] Hawton K, Zahl D, Weatherall R (2003) Suicide following deliberate self-harm: long-term follow-up of patients who presented to a general hospital. Br J Psychiatry 182: 537–542. 12777346 10.1192/bjp.182.6.537

[4] Sinclair JM, Gray A, Rivero-Arias O, Saunders KE, Hawton K (2011) Healthcare and social services resource use and costs of self-harm patients. Soc Psychiatry Psychiatr Epidemiol 46: 263–271. 20174782 10.1007/s00127-010-0183-5

[5] Zahl DL, Hawton K (2004) Repetition of deliberate self-harm and subsequent suicide risk: long-term follow-up study of 11,583 patients. Br J Psychiatry 185: 70–75. 15231558 10.1192/bjp.185.1.70

[6] Owens D, Horrocks J, House A (2002) Fatal and non-fatal repetition of self-harm. Systematic review. Br J Psychiatry 181: 193–199. 12204922 10.1192/bjp.181.3.193

[7] Ito T, Hada M, Kimura A, Kurosawa H, Okubo Y (2004) The study of suicide attempters in a critical care medical center and their prognosis. Seishin Igaku 46: 389–396.

[8] Chen VC, Tan HK, Cheng AT, Chen CY, Liao LR, et al (2010) Non-fatal repetition of self-harm: population-based prospective cohort study in Taiwan. British Journal of Psychiatry 196: 31–35. 20044656 10.1192/bjp.bp.109.067009

[9] Yip PS, Hawton K, Liu K, Liu KS, Ng PW, et al (2011) A study of deliberate self-harm and its repetition among patients presenting to an emergency department. Crisis 32: 217–224. 21940251 10.1027/0227-5910/a000069

[10] Lee Y, Lin PY, Yeh WC, Chiu NM, Hung CF, et al (2012) Repeated suicide attempts among suicidal cases: Outcome of one-year follow-up. Asia-Pacific Psychiatry 4: 174–180.

[11] Hassanian-Moghaddam H, Sarjami S, Kolahi AA, Carter GL (2011) Postcards in Persia: randomised controlled trial to reduce suicidal behaviours 12 months after hospital-treated self-poisoning. Br J Psychiatry 198: 309–316. 21343332 10.1192/bjp.bp.109.067199

[12] Chung CH, Lai CH, Chu CM, Pai L, Kao S, et al (2012) A nationwide, population-based, long-term follow-up study of repeated self-harm in Taiwan. BMC Public Health 12: 744. 22950416 10.1186/1471-2458-12-744 PMC3488309

[13] Liu L, Xiao S (2002) A follow-up study of suicide attempters. Chinese Mental Health Journal 16: 253–256.

[14] Morthorst B, Krogh J, Erlangsen A, Alberdi F, Nordentoft M (2012) Effect of assertive outreach after suicide attempt in the AID (assertive intervention for deliberate self harm) trial: randomised controlled trial. BMJ 345: e4972. 22915730 10.1136/bmj.e4972 PMC3425442

[15] Schmidtke A, Bille-Brahe U, DeLeo D, Kerkhof A, Bjerke T, et al (1996) Attempted suicide in Europe: Rates, trends and sociodemographic characteristics of suicide attempters during the period 1989–1992. Results of the WHO/EURO Multicentre Study on Parasuicide. Acta Psychiatrica Scandinavica 93: 327–338. 8792901 10.1111/j.1600-0447.1996.tb10656.x

[16] Rucker G, Schwarzer G, Carpenter JR, Schumacher M (2008) Undue reliance on I(2) in assessing heterogeneity may mislead. BMC Med Res Methodol 8: 79. 19036172 10.1186/1471-2288-8-79 PMC2648991

[17] Lipsey MW, Wilson BW (2001) Practical meta-analysis. Thousand Oaks SAGE: Calif.

[18] Higgins JP, Thompson SG (2004) Controlling the risk of spurious findings from meta-regression. Stat Med 23: 1663–1682. 15160401 10.1002/sim.1752

[19] Higgins JP (2008) Commentary: Heterogeneity in meta-analysis should be expected and appropriately quantified. Int J Epidemiol 37: 1158–1160. 18832388 10.1093/ije/dyn204

[20] Gunnell D, Bennewith O, Peters TJ, House A, Hawton K (2005) The epidemiology and management of self-harm amongst adults in England. J Public Health (Oxf) 27: 67–73. 15564277 10.1093/pubmed/fdh192

[21] Bennewith O, Gunnell D, Peters T, Hawton K, House A (2004) Variations in the hospital management of self harm in adults in England: observational study. BMJ 328: 1108–1109. 15130979 10.1136/bmj.328.7448.1108 PMC406323

[22] Gunnell D, Eddleston M (2003) Suicide by intentional ingestion of pesticides: a continuing tragedy in developing countries. Int J Epidemiol 32: 902–909. 14681240 10.1093/ije/dyg307 PMC2001280

[23] Eddleston M, Gunnell D, Karunaratne A, de Silva D, Sheriff MH, et al (2005) Epidemiology of intentional self-poisoning in rural Sri Lanka. Br J Psychiatry 187: 583–584. 16319413 10.1192/bjp.187.6.583 PMC1475924

[24] Mohamed F, Perera A, Wijayaweera K, Kularatne K, Jayamanne S, et al (2011) The prevalence of previous self-harm amongst self-poisoning patients in Sri Lanka. Soc Psychiatry Psychiatr Epidemiol 46: 517–520. 20372876 10.1007/s00127-010-0217-z PMC3092923

[25] Gilbody S, House A, Owens D (1997) The early repetition of deliberate self harm. J R Coll Physicians Lond 31: 171–172. 9131517 PMC5420906

[26] Horrocks J, Price S, House A, Owens D (2003) Self-injury attendances in the accident and emergency department: Clinical database study. British Journal of Psychiatry 183: 34–39. 12835241 10.1192/bjp.183.1.34

[27] Runeson B, Tidemalm D, Dahlin M, Lichtenstein P, Langstrom N (2010) Method of attempted suicide as predictor of subsequent successful suicide: national long term cohort study. BMJ 341: c3222. 20627975 10.1136/bmj.c3222 PMC2903664

[28] Bergen H, Hawton K, Waters K, Ness J, Cooper J, et al (2012) How do methods of non-fatal self-harm relate to eventual suicide? Journal of Affective Disorders 136: 526–533. 22127391 10.1016/j.jad.2011.10.036

