# Supplementary material for: Hospital Presenting Self-Harm and Risk of Fatal and Non-Fatal Repetition: Systematic Review and Meta-Analysis
Source: PLoS One. 2014 Feb 28;9(2):e89944. doi: 10.1371/journal.pone.0089944 (PMC3938547; doi:10.1371/journal.pone.0089944)
Supplement: Appendix S1 — Search strategy. (DOCX) [file pone.0089944.s001.docx]

Appendix S1.

Search strategy:

1. suicide/ or suicide, attempted / or self-injurious behaviour/ or suicidal behaviour/ or automutilation/ or drug overdose/ or attempted suicide/ or self destructive behaviour/ or self inflicted wounds/ or self mutilation/ or drug overdoses/ or suicidal ideation/ or injuries, self inflicted/

2. (self-harm$ or self?harm$ or self-injur$ or self?injur$ or self-mutilat$ or self?mutilat$ or suicid$ or self-destruct$ or self?destruct$ or self-poison$ or self?poison$ or (self adj2 cut$) or self-immolat$ or self?immolat$ or self-inflict$ or self?inflict$ or auto-mutilat$ or auto mutilat$ or overdose$).tw.

3. 1 or 2

4. (review or case report or case reports or news or comparative study or letter or comment).pt.

5. chemotherapy/ or cell line, tumor/ or (suicide adj gene).tw

6. suicide adj5 vector.tw

7. Cancer.mp

8. 3 not (4 or 5 or 6 or 7)

9. (hospital$ or hospital treat$ or (accident adj3 department) or emergency or general practice or general hospital or psychiatric hospital$ or medically treated or repeat$ or repetit$ or admit$ or follow?up or follow-up or prospective$ or epidemiolog$).tw.

10. (epidemiology or cohort or follow?up or hospital readmission).sh

11. 8 and (9 or 10)

12. ("2000" or "2001" or "2002" or "2003" or "2004" or "2005" or "2006" or "2007" or "2008" or "2009" or "2010" or "2011" or "2012").yr.

13. 11 and 12

14. animal welfare/ or animals/ or laws/or professional ethics/ or assisted suicide/or right to die.sh

15. book.pt.

16. (case report$ or hunger strike or case series or qualitative).tw

17. (comment or reply or book or letter or bereavement) .tw

18. qualitative study.mp

19. 13 not (or/17-18)

20. (bipolar or (anxiety adj3 disorder) or personality disorder$ or borderline personality or schizophren$ or depress$ or alcohol depend$).tw

21. 19 not 20

22. (child$ or adolesc$ or boy$ or girl$ youth$ or preadolesc$ or school or juvenile$ ).tw.

23. (elderly or old$ or geriatric).tw.

24. 21 not (22 or 23)
