# Supplementary material for: Hospital Presenting Self-Harm and Risk of Fatal and Non-Fatal Repetition: Systematic Review and Meta-Analysis
Source: PLoS One. 2014 Feb 28;9(2):e89944. doi: 10.1371/journal.pone.0089944 (PMC3938547; doi:10.1371/journal.pone.0089944)
Supplement: Appendix S2 — Characteristic of included studies. (DOCX) [file pone.0089944.s002.docx]

Appendix S2.

| Table 1. Characteristics of included studies* | | | | | | |
| --- | --- | --- | --- | --- | --- | --- |
| Author** | Year published | Design | Country | n | Follow-up (Yrs) | % male |
| Buglass[[1](#_ENREF_1)] | 1970 | Cohort | United Kingdom | 511 | 3 | 34.2 |
| Rosen[[2](#_ENREF_2)] | 1970 | Cohort | United Kingdom | 886 | 1 | 40 |
| Bratfos[[3](#_ENREF_3)] | 1971 | Cohort | Norway | 316 | 8 | 41.5 |
| Greer[[4](#_ENREF_4)] | 1971 | Cohort | United Kingdom | 211 | 1.5 | 35.6 |
| Pederson[[5](#_ENREF_5)] | 1973 | Cohort | United States | 1345 | 2.5 | 25.7 |
| Buglass[[6](#_ENREF_6)] | 1974 | Cohort | United Kingdom | 2809 | 1 | - |
| Paerregaard[[7](#_ENREF_7)] | 1975 | Cohort | Denmark | 484 | 10.5 | 40.9 |
| Lonnqvist[[8](#_ENREF_8)] | 1975 | Cohort | Finland | 100 | 8 | 28 |
| Garzotto[[9](#_ENREF_9)] | 1976 | Cohort | Italy | 120 | 1 | - |
| Morgan[[10](#_ENREF_10)] | 1976 | Cohort | United Kingdom | 279 | 1.5 | 36.9 |
| Gharagozlou[[11](#_ENREF_11)] | 1977 | Cohort | Iran | 100 | 3 | - |
| Gardner[[12](#_ENREF_12)] | 1977 | RCT | United Kingdom | 246 | 1 | - |
| Bancroft[[13](#_ENREF_13)] | 1977 | Cohort | United Kingdom | 690 | 2 | 32.6 |
| Gibbons[[14](#_ENREF_14)] | 1978 | RCT | United Kingdom | 200 | 1 | - |
| Siani[[15](#_ENREF_15)] | 1979 | Cohort | Italy | 147 | 1 | - |
| Pierce[[16](#_ENREF_16)] | 1981 | Cohort | United Kingdom | 500 | 5 | - |
| Adam[[17](#_ENREF_17)] | 1981 | Cohort | New Zealand | 195 | 3 | - |
| Gardner[[18](#_ENREF_18)] | 1982 | Cohort | United Kingdom | 213 | 1 | - |
| Rosenman[[19](#_ENREF_19)] | 1983 | Cohort | Australia | 262 | 5 | 33 |
| Adam[[20](#_ENREF_20)] | 1983 | Cohort | New Zealand | 98 | 21 | - |
| Hansen[[21](#_ENREF_21)] | 1984 | Cohort | Denmark | 208 | 1.9 | 54.8 |
| Pierce[[22](#_ENREF_22)] | 1984 | Cohort | United Kingdom | 500 | 5 | 34 |
| Pallis[[23](#_ENREF_23)] | 1984 | Cohort | United Kingdom | 1263 | 2 | - |
| Lonnqvist[[24](#_ENREF_24)] | 1984 | Cohort | Finland | 209 | 6 | 41.6 |
| McFarland[[25](#_ENREF_25)] | 1986 | Cohort | United States | 282 | 1 | 51.4 |
| Wilkinson[[26](#_ENREF_26)] | 1987 | Cohort | United Kingdom | 1376 | 1.5 | 40 |
| Sundqvist-Stensman[[27](#_ENREF_27)] | 1988 | Cohort | Sweden | 1273 | 4 | 48.3 |
| Steer[[28](#_ENREF_28)] | 1988 | Cohort | United States | 499 | 7.5 | 42.5 |
| Hawton[[29](#_ENREF_29)] | 1988 | Cohort | United Kingdom | 1959 | 8.3 | 33 |
| Culberg[[30](#_ENREF_30)] | 1988 | Cohort | Sweden | 163 | 9 | 42 |
| Rygnestad[[31](#_ENREF_31)] | 1988 | Cohort | Norway | 253 | 5 | 40.3 |
| Hassanyeh[[32](#_ENREF_32)] | 1989 | Cohort | United Kingdom | 98 | 1.5 | - |
| Hawton[[33](#_ENREF_33)] | 1989 | Cohort | United Kingdom | 4371 | 5 | 34.8 |
| Moller[[34](#_ENREF_34)] | 1989 | RCT | Germany | 85 | 1 | - |
| Sakinofsky[[35](#_ENREF_35)] | 1990 | Cohort | Canada | 228 | 1 | 35.3 |
| Allgulander[[36](#_ENREF_36)] | 1990 | Cohort | Sweden | 8895 | 6 | 38 |
| Nielsen[[37](#_ENREF_37)] | 1990 | Cohort | Denmark | 207 | 5 | 51.7 |
| Lonnqvist[[38](#_ENREF_38)] | 1991 | Cohort | Finland | 1600 | 4.5 | 42.4 |
| Suokas[[39](#_ENREF_39)] | 1991 | Cohort | Finland | 1018 | 5.5 | 47 |
| Ekeberg[[40](#_ENREF_40)] | 1991 | Cohort | Norway | 934 | 5 | 49.7 |
| Ojehagen[[41](#_ENREF_41)] | 1992 | Cohort | Sweden | 237 | 1 | 41 |
| Allard[[42](#_ENREF_42)] | 1992 | RCT | Canada | 74 | 2 | 46 |
| Morgan[[43](#_ENREF_43)] | 1993 | RCT | United Kingdom | 111 | 1 | - |
| Nordentoft[[44](#_ENREF_44)] | 1993 | Cohort | Denmark | 974 | 8.48 | 42.3 |
| Bille-Brahe[[45](#_ENREF_45)] | 1994 | Cohort | Denmark | 773 | 2 | 42 |
| Ekeberg[[46](#_ENREF_46)] | 1994 | Cohort | Norway | 926 | 10 | 49.7 |
| Boyes[[47](#_ENREF_47)] | 1994 | Cohort | United Kingdom | 1597 | 2.5 | 40.6 |
| Van Heeringen[[48](#_ENREF_48)] | 1995 | RCT | Belgium | 258 | 1 | 48 |
| Arensman[[49](#_ENREF_49)] | 1995 | Cohort | Netherlands | 690 | 2 | 37.1 |
| Nordstrom[[50](#_ENREF_50)] | 1995 | Cohort | Sweden | 1573 | 5 | 36.2 |
| De Moore[[51](#_ENREF_51)] | 1996 | Cohort | Australia | 223 | 18 | 32.7 |
| Johnsson[[52](#_ENREF_52)] | 1996 | Cohort | Sweden | 75 | 1 | 41.3 |
| Hjelmeland[[53](#_ENREF_53)] | 1996 | Cohort | Norway | 1016 | 1 | 40 |
| Palsson[[54](#_ENREF_54)] | 1996 | Cohort | Iceland | 307 | 7.4 | 40.7 |
| Schmidtke[[55](#_ENREF_55)] | 1996 | Cohort | Multi-countryƗ | 16394 | 2 | 40 |
| Van Der Sande[[56](#_ENREF_56)] | 1997 | RCT | Netherlands | 134 | 1 | 34 |
| McEvedy[[57](#_ENREF_57)] | 1997 | Cohort | United Kingdom | 628 | 2 | - |
| Gilbody[[58](#_ENREF_58)] | 1997 | Cohort | United Kingdom | 1576 | 1 | 44 |
| Hawton[[59](#_ENREF_59)] | 1997 | Cohort | United Kingdom | 3644 | 1 | 39.4 |
| Hjelmeland[[60](#_ENREF_60)] | 1998 | Cohort | Multi-countryƗ | 776 | 1 | 39 |
| Batt[[61](#_ENREF_61)] | 1998 | Cohort | France | 632 | 0.5 | 37 |
| Hall[[62](#_ENREF_62)] | 1998 | Cohort | United Kingdom | 8304 | 13.5 | 40.3 |
| Crawford[[63](#_ENREF_63)] | 1998 | Cohort | United Kingdom | 294 | 1.5 | - |
| Tejedor[[64](#_ENREF_64)] | 1999 | Cohort | Spain | 150 | 10 | 44 |
| Salander Renberg[[65](#_ENREF_65)] | 1999 | Cohort | Sweden | 1194 | 3 | 40 |
| Curran[[66](#_ENREF_66)] | 1999 | Cohort | Ireland | 85 | 8.5 | 38 |
| Kennedy[[67](#_ENREF_67)] | 1999 | Cohort | United Kingdom | 137 | 21 | 49.6 |
| Ruchholtz[[68](#_ENREF_68)] | 1999 | Cohort | Germany | 65 | 6.1 | 57 |
| Cusick[[69](#_ENREF_69)] | 1999 | Cohort | United States | 52 | 4.5 | 78 |
| Carter[[70](#_ENREF_70)] | 1999 | Cohort | Australia | 1238 | 1 | - |
| Gonzalez[[71](#_ENREF_71)] | 2000 | Cohort | Spain | 167 | 0.5 | 27 |
| Dieserud[[72](#_ENREF_72)] | 2000 | Cohort | Norway | 1031 | 6 | 36 |
| Evans[[73](#_ENREF_73)] | 2000 | Cohort | United Kingdom | 467 | 1 | 46.7 |
| Archinard[[74](#_ENREF_74)] | 2000 | Cohort | Switzerland | 59 | 2 | - |
| Morgan[[75](#_ENREF_75)] | 2000 | Cohort | United Kingdom | 142 | 1 | 41.5 |
| Scocco[[76](#_ENREF_76)] | 2000 | Cohort | Italy | 421 | 3.5 | 30 |
| Engeland[[77](#_ENREF_77)] | 2001 | Cohort | Norway | 271 | 5.5 | 52 |
| Suokas[[78](#_ENREF_78)] | 2001 | Cohort | Finland | 1018 | 14 | 47 |
| Abumadini[[79](#_ENREF_79)] | 2001 | Cohort | Saudi Arabia | 362 | 3 | 35.6 |
| Guthrie[[80](#_ENREF_80)] | 2001 | RCT | United Kingdom | 61 | 0.5 | 45.9 |
| Ostamo[[81](#_ENREF_81)] | 2001 | Cohort | Finland | 2782 | 5.3 | 50.4 |
| Carter[[82](#_ENREF_82)] | 2002 | Cohort | Australia | 1317 | 1 | 37.7 |
| Nimeus[[83](#_ENREF_83)] | 2002 | Cohort | Sweden | 555 | 4.5 | 37.3 |
| Bennewith[[84](#_ENREF_84)] | 2002 | RCT | United Kingdom | 968 | 1 | 42.7 |
| Antretter[[85](#_ENREF_85)] | 2002 | Cohort | Austria | 137 | 1 | 43.8 |
| Liu[[86](#_ENREF_86)] | 2002 | Cohort | China | 100 | 1 | 21.7 |
| Jenkins[[87](#_ENREF_87)] | 2002 | Cohort | United Kingdom | 180 | 21.75 | 31 |
| Clarke[[88](#_ENREF_88)] | 2002 | RCT | United Kingdom | 247 | 1 | 45 |
| Hawton[[89](#_ENREF_89)] | 2003 | Cohort | United Kingdom | 2379 | 1 | 40.9 |
| Kapur[[90](#_ENREF_90)] | 2003 | Cohort | United Kingdom | 1306 | 0.23 | 45.6 |
| Hawton[[91](#_ENREF_91)] | 2003 | Cohort | United Kingdom | 11583 | 11.5 | 39.8 |
| Moron[[92](#_ENREF_92)] | 2003 | Cohort | Spain | 467 | 4.5 | 35.1 |
| Keeley[[93](#_ENREF_93)] | 2003 | Cohort | Ireland | 2287 | 1 | 46 |
| Tyrer[[94](#_ENREF_94)] | 2003 | RCT | United Kingdom | 241 | 1 | 32 |
| Horrocks[[95](#_ENREF_95)] | 2003 | Cohort | United Kingdom | 3167 | 1.5 | 47.2 |
| Monti[[96](#_ENREF_96)] | 2003 | Cohort | Sweden | 97 | 0.25 | 27 |
| Dieserud[[97](#_ENREF_97)] | 2003 | Cohort | Norway | 50 | 1.5 | 33 |
| Suominen[[98](#_ENREF_98)] | 2004 | Cohort | Finland | 1198 | 5 | 47.5 |
| Courtet[[99](#_ENREF_99)] | 2004 | Cohort | France | 103 | 1 | 17.5 |
| Henriques[[100](#_ENREF_100)] | 2004 | Cohort | United States | 258 | 1 | 42.6 |
| Corcoran[[101](#_ENREF_101)] | 2004 | Cohort | Ireland | 3325 | 1.5 | 45 |
| Soderberg[[102](#_ENREF_102)] | 2004 | Cohort | Sweden | 63 | 7.5 | 37.3 |
| Ito[[103](#_ENREF_103)] | 2004 | Cohort | Japan | 103 | 2 | 35 |
| Beautrais[[104](#_ENREF_104)] | 2004 | Cohort | New Zealand | 302 | 5 | 46.4 |
| Suominen[[105](#_ENREF_105)] | 2004 | Cohort | Finland | 224 | 12 | 44.2 |
| Reith[[106](#_ENREF_106)] | 2004 | Cohort | Australia | 4105 | - | 42.3 |
| Suominen[[107](#_ENREF_107)] | 2004 | Cohort | Finland | 100 | 37 | 28 |
| Skogman[[108](#_ENREF_108)] | 2004 | Cohort | Sweden | 1052 | 6.42 | 39 |
| Brown[[109](#_ENREF_109)] | 2005 | RCT | United States | 60 | 1.5 | 38.3 |
| Evans[[110](#_ENREF_110)] | 2005 | RCT | United Kingdom | 410 | 1 | - |
| Carter[[111](#_ENREF_111)] | 2005 | RCT | Australia | 394 | 1 | 26 |
| Gibb[[112](#_ENREF_112)] | 2005 | Cohort | New Zealand | 3690 | 5 | 39.8 |
| Harriss[[113](#_ENREF_113)] | 2005 | Cohort | United Kingdom | 2719 | 5.2 | 41.8 |
| Leslie[[114](#_ENREF_114)] | 2005 | Cohort | United Kingdom | 3018 | 2.75 | 44 |
| Owens[[115](#_ENREF_115)] | 2005 | Cohort | United Kingdom | 976 | 16.75 | 39.4 |
| Kapur[[116](#_ENREF_116)] | 2006 | Cohort | United Kingdom | 9213 | 1 | 43.3 |
| Benjaminsen[[117](#_ENREF_117)] | 2006 | Cohort | Denmark | 126 | 1 | 37 |
| Wang[[118](#_ENREF_118)] | 2006 | Cohort | Denmark | 125 | 20 | 46.4 |
| Tiihonen[[119](#_ENREF_119)] | 2006 | Cohort | Finland | 15390 | 3.4 | 48.5 |
| Johnston[[120](#_ENREF_120)] | 2006 | Cohort | United Kingdom | 4743 | 0.5 | - |
| Pulido[[121](#_ENREF_121)] | 2006 | Cohort | Spain | 1331 | 2.5 | 33 |
| Vaiva[[122](#_ENREF_122)] | 2006 | RCT | France | 312 | 1.08 | 29 |
| Belgamwar[[123](#_ENREF_123)] | 2006 | Cohort | United Kingdom | 6317 | 4 | 46.5 |
| Eudier[[124](#_ENREF_124)] | 2006 | Cohort | France | 1003 | 1 | 41.2 |
| Carter[[125](#_ENREF_125)] | 2007 | RCT | Australia | 394 | 2 | 26 |
| Loas[[126](#_ENREF_126)] | 2007 | Cohort | France | 167 | 36.5 | 23.6 |
| Haw[[127](#_ENREF_127)] | 2007 | Cohort | United Kingdom | 4167 | 5.5 | 43.8 |
| Christiansen[[128](#_ENREF_128)] | 2007 | Cohort | Denmark | 3614 | 3.88 | 41.2 |
| Lindqvist[[129](#_ENREF_129)] | 2007 | Cohort | Sweden | 196 | 8.2 | 44 |
| Caldera[[130](#_ENREF_130)] | 2007 | Cohort | Nicaragua | 204 | 3.2 | 24.5 |
| Haukka[[131](#_ENREF_131)] | 2008 | Cohort | Finland | 18199 | 3.6 | 49 |
| Howsen[[132](#_ENREF_132)] | 2008 | Cohort | New Zealand | 754 | 1 | - |
| Chandrasekaran[[133](#_ENREF_133)] | 2008 | Cohort | India | 341 | 2 | 43.7 |
| McAuliffe[[134](#_ENREF_134)] | 2008 | Cohort | Ireland | 152 | 1 | 37.5 |
| Payne[[135](#_ENREF_135)] | 2009 | Cohort | United Kingdom | 50891 | 2 | 43 |
| Hvid[[136](#_ENREF_136)] | 2009 | Cohort | Denmark | 58 | 1 | 31 |
| Heyerdahl[[137](#_ENREF_137)] | 2009 | Cohort | Norway | 2062 | 1 | 60 |
| Nakagawa[[138](#_ENREF_138)] | 2009 | Cohort | Japan | 144 | 1.75 | - |
| Antretter[[139](#_ENREF_139)] | 2009 | Cohort | Austria | 4140 | 10 | 45.4 |
| Prescott[[140](#_ENREF_140)] | 2009 | Cohort | United Kingdom | 1598 | 1 | 40.8 |
| Scoliers[[141](#_ENREF_141)] | 2009 | Cohort | Belgium | 361 | 5 | 43.5 |
| Bjornaas[[142](#_ENREF_142)] | 2009 | Cohort | Norway | 946 | 20 | 49 |
| Bergen[[143](#_ENREF_143)] | 2010 | Cohort | United Kingdom | 7394 | 1 | 40.2 |
| Waren[[144](#_ENREF_144)] | 2010 | Cohort | Sweden | 162 | - | 21.8 |
| Crawford[[145](#_ENREF_145)] | 2010 | RCT | United Kingdom | 52 | 0.5 | 51.9 |
| Mehlum[[146](#_ENREF_146)] | 2010 | Cohort | Norway | 569 | 3.4 | 34.8 |
| Bergen[[147](#_ENREF_147)] | 2010 | Cohort | United Kingdom | 13996 | 2 | 41.8 |
| Onen Sertoz[[148](#_ENREF_148)] | 2010 | Cohort | Hungary | 55 | 0.5 | 20 |
| Chen[[149](#_ENREF_149)] | 2010 | Cohort | Taiwan | 970 | 3.8 | 37 |
| Beautrais[[150](#_ENREF_150)] | 2010 | RCT | New Zealand | 174 | 1 | 38 |
| Russell[[151](#_ENREF_151)] | 2010 | Cohort | United Kingdom | 675 | 1.75 | 43.6 |
| Sverrisson[[152](#_ENREF_152)] | 2010 | Cohort | Iceland | 224 | 4.1 | 39 |
| Bertolote[[153](#_ENREF_153)] | 2010 | RCT | Multi-countryƗ | 57Ɨ | 1.5 | 33 |
| Runeson[[154](#_ENREF_154)] | 2010 | Cohort | Sweden | 48649 | 26 | 48 |
| Johannessen[[155](#_ENREF_155)] | 2011 | Cohort | Norway | 1616 | 1 | 31.7 |
| Hatcher[[156](#_ENREF_156)] | 2011 | RCT | New Zealand | 572 | 1 | 33.9 |
| Lee[[157](#_ENREF_157)] | 2011 | Cohort | Taiwan | 145 | 1 | 31.4 |
| Karasouli[[158](#_ENREF_158)] | 2011 | Cohort | United Kingdom | 976 | 17 | 39 |
| Hvid[[159](#_ENREF_159)] | 2011 | RCT | Denmark | 64 | 1 | 28 |
| Maier[[160](#_ENREF_160)] | 2011 | Cohort | Switzerland | 190 | 6 | 32 |
| Yip[[161](#_ENREF_161)] | 2011 | Cohort | China | 92 | 0.5 | 29 |
| Hassanian-Moghaddam[[162](#_ENREF_162)] | 2011 | RCT | Iran | 1150 | 1 | 34 |
| Chung[[163](#_ENREF_163)] | 2012 | Cohort | Taiwan | 39875 | 4.5 | 46.1 |
| Kuo[[164](#_ENREF_164)] | 2012 | Cohort | Taiwan | 7601 | 3.3 | 30.5 |
| Cebria[[165](#_ENREF_165)] | 2012 | Cohort | Spain | 387 | 1 | 32.8 |
| Monnin[[166](#_ENREF_166)] | 2012 | Cohort | France | 273 | 2 | 30.8 |
| Sjostrom[[167](#_ENREF_167)] | 2012 | Cohort | Sweden | 155 | 3 | 20 |
| Perry[[168](#_ENREF_168)] | 2012 | Cohort | Ireland | 48206 | 3.5 | 44 |
| Stefansson[[169](#_ENREF_169)] | 2012 | Cohort | Sweden | 81 | 12.5 | 43 |
| Bilen[[170](#_ENREF_170)] | 2012 | Cohort | Sweden | 1524 | 2.5 | 35 |
| Morthorst[[171](#_ENREF_171)] | 2012 | RCT | Denmark | 120 | 1 | 27 |
| Isung[[172](#_ENREF_172)] | 2012 | Cohort | Sweden | 58 | 13 | 39.7 |
| Riedi[[173](#_ENREF_173)] | 2012 | Cohort | France | 184 | 0.5 | 29 |
| Pan[[174](#_ENREF_174)] | 2012 | Cohort | Taiwan | 50805 | 1.28 | 33.5 |
| Bergen[[175](#_ENREF_175)] | 2012 | Cohort | United Kingdom | 30202 | 7 | 41.4 |
| Choi[[176](#_ENREF_176)] | 2012 | Cohort | Korea, South | 349 | 1.77 | 25.2 |
| Jimenez Trevino[[177](#_ENREF_177)] | 2012 | Cohort | Spain | 279 | 0.5 | 38.6 |

Footnote for table: * A number of studies reported on multiple cohorts/time points/centres, the first cohort/time point/centre recorded from the paper is described here. ** Full reference included below. Ɨ Multi-country study details: Schmidtke 1996 – multiple countries from the WHO/EURO study and reported one overall estimate of repetition; Hjelmeland 1998 - included 5 Nordic regions of the WHO/EURO study and reported one overall estimate of repetition; Bertolote 2010 – included centres in Brazil (n=71), India (n=357), Sri Lanka (n=149), Iran (n=311) and China (described in table).

References of Included papers:

1. Buglass D, McCulloch JW (1970) Further suicidal behaviour: the development and validation of predictive scales. British Journal of Psychiatry 116: 483-491.

2. Rosen DH (1970) The serious suicide attempt: epidemiological and follow-up study of 886 patients. Am J Psychiatry 127: 764-770.

3. Bratfos O (1971) Attempted suicide. A comparative study of patients who have attempted suicide and psychiatric patients in general. Acta Psychiatr Scand 47: 38-56.

4. Greer S, Bagley C (1971) Effect of psychiatric intervention in attempted suicide: a controlled study. Br Med J 1: 310-312.

5. Pederson AM, Awad GA, Kindler AR (1973) Epidemiological differences between white and nonwhite suicide attempters. Am J Psychiatry 130: 1071-1076.

6. Buglass D, Horton J (1974) The repetition of parasuicide: a comparison of three cohorts. Br J Psychiatry 125: 168-174.

7. Paerregaard G (1975) Suicide among attempted suicdes: a 10-year follow-up. Suicide 5: 140-144.

8. Lonnqvist J, Niskanen P, Achte K, Ginman L (1975) Self-poisoning with follow-up considerations. Suicide 5: 39-46.

9. Garzotto N, Siani R, Tansella CZ, Tansella M (1976) Cross-validation of a predictive scale for subsequent suicidal behaviour in an Italian sample. Br J Psychiatry 128: 137-140.

10. Morgan HG, Barton J, Pottle S, Pocock H, Burns-Cox CJ (1976) Deliberate self-harm: a follow-up study of 279 patients. Br J Psychiatry 128: 361-368.

11. Gharagozlou H, Hadjmohammadi M (1977) Report on a three-year follow-up of 100 cases of suicidal attempts in Shiraz, Iran. Int J Soc Psychiatry 23: 209-210.

12. Gardner R, Hanka R, O'Brien VC, Page AJ, Rees R (1977) Psychological and social evaluation in cases of deliberate self-poisoning admitted to a general hospital. Br Med J 2: 1567-1570.

13. Bancroft J, Marsack P (1977) The repetitiveness of self-poisoning and self-injury. Br J Psychiatry 131: 394-399.

14. Gibbons JS, Butler J, Urwin P, Gibbons JL (1978) Evaluation of a social work service for self-poisoning patients. Br J Psychiatry 133: 111-118.

15. Siani R, Garzotto N, Tansella CZ, Tansella M (1979) Predictive scales for parasuicide repetition. Further results. Acta Psychiatr Scand 59: 17-23.

16. Pierce DW (1981) The predictive validation of a suicide intent scale: a five year follow-up. Br J Psychiatry 139: 391-396.

17. Adam KS, Isherwood J, Taylor G, Scarr G, Streiner DL (1981) Attempted suicide in Christchurch: three-year follow-up of 195 patients. N Z Med J 93: 376-381.

18. Gardner R, Hanka R, Roberts SJ, Allon-Smith JM, Kings AA, et al. (1982) Psychological and social evaluation in cases of deliberate self-poisoning seen in an accident department. Br Med J (Clin Res Ed) 284: 491-493.

19. Rosenman SJ (1983) Subsequent deaths after attempted suicide by drug overdose in the western region of Adelaide, 1976. Med J Aust 2: 496-499.

20. Adam KS, Valentine J, Scarr G, Streiner D (1983) Follow-up of attempted suicide in Christchurch. Aust N Z J Psychiatry 17: 18-25.

21. Hansen W, Wang AG (1984) Suicide attempts in a Danish region. Soc Psychiatry 19: 197-201.

22. Pierce D (1984) Suicidal intent and repeated self-harm. Psychol Med 14: 655-659.

23. Pallis DJ, Gibbons JS, Pierce DW (1984) Estimating suicide risk among attempted suicides. II. Efficiency of predictive scales after the attempt. Br J Psychiatry 144: 139-148.

24. Lonnqvist J, Karha E (1984) Suicide attempts in Helsinki. Psychiatria Fennica 15: 135-145.

25. McFarland BH, Beavers DJ (1986) Psychiatric consultation following attempted suicide. J Am Osteopath Assoc 86: 743-750.

26. Wilkinson G, Smeeton N (1987) The repetition of parasuicide in Edinburgh 1980-1981. Soc Psychiatry 22: 14-19.

27. Sundqvist-Stensman U (1988) Suicides among persons treated for self-poisoning at an ICU. Opuscula Medica 33: 71-76.

28. Steer R, Beck A, Garrison B, Lester D (1988) Eventual suicide in interrupted and uninterrupted attempters: A challenge to the cry-for-help hypothesis. Suicide and Life-Threatening Behavior 18: 119-128.

29. Hawton K, Fagg J (1988) Suicide, and other causes of death, following attempted suicide. Br J Psychiatry 152: 359-366.

30. Cullberg J, Wasserman D, Stefansson C, G. (1988) Who commits suicide after a suicide attempt? An 8 to 10 year follow up in a suburban catchment area. Acta Psychiatrica Scandinavica 77: 598-603.

31. Rygnestad T (1988) A prospective 5-year follow-up study of self-poisoned patients. Acta Psychiatr Scand 77: 328-331.

32. Hassanyeh F, O'Brien G, Holton AR, Hurren K, Watt L (1989) Repeat self-harm: an 18-month follow-up. Acta Psychiatr Scand 79: 265-267.

33. Hawton K, Fagg J, McKeown SP (1989) Alcoholism, alcohol and attempted suicide. Alcohol Alcohol 24: 3-9.

34. Moller HJ (1989) Efficacy of different strategies of aftercare for patients who have attempted suicide. J R Soc Med 82: 643-647.

35. Sakinofsky I, Roberts RS, Brown Y, Cumming C, James P (1990) Problem resolution and repetition of parasuicide. A prospective study. British Journal of Psychiatry 156: 395-399.

36. Allgulander C, Fisher LD (1990) Clinical predictors of completed suicide and repeated self-poisoning in 8895 self-poisoning patients. Eur Arch Psychiatry Neurol Sci 239: 270-276.

37. Nielsen B, Wang A, Brille-Brahe U (1990) Attempted suicide in Denmark. IV. A five-year follow up. Acta Psychiatrica Scandinavica 81: 250-254.

38. Lonnqvist J, Ostamo A (1991) Suicide following the first suicide attempt: a five year follow-up using a survival analysis. Psychiatria Fennica 22: 171-179.

39. Suokas J, Lonnqvist J (1991) Outcome of attempted suicide and psychiatric consultation: risk factors and suicide mortality during a five-year follow-up. Acta Psychiatr Scand 84: 545-549.

40. Ekeberg O, Ellingsen O, Jacobsen D (1991) Suicide and other causes of death in a five-year follow-up of patients treated for self-poisoning in Oslo. Acta Psychiatr Scand 83: 432-437.

41. Ojehagen A, Danielsson M, Traskman-Bendz L (1992) Deliberate self-poisoning: Treatment follow-up of repeaters and nonrepeaters. Acta Psychiatrica Scandinavica 85: 370-375.

42. Allard R, Marshall M, Plante MC (1992) Intensive follow-up does not decrease the risk of repeat suicide attempts. Suicide Life Threat Behav 22: 303-314.

43. Morgan HG, Jones EM, Owen JH (1993) Secondary prevention of non-fatal deliberate self-harm. The green card study. Br J Psychiatry 163: 111-112.

44. Nordentoft M, Breum L, Munck LK, Nordestgaard AG, Hunding A, et al. (1993) High mortality by natural and unnatural causes: a 10 year follow up study of patients admitted to a poisoning treatment centre after suicide attempts. BMJ 306: 1637-1641.

45. Bille-Brahe U, Jessen G (1994) Repeated suicidal behavior: a two-year follow-up. Crisis: Journal of Crisis Intervention & Suicide 15: 77-82.

46. Ekeberg O, Ellingsen O, Jacobsen D (1994) Mortality and causes of death in a 10-year follow-up study of patients treated for self-poisoning in Oslo. Suicide and Life-Threatening Behavior 24: 398-405.

47. Boyes AP (1994) Repetition of overdose: a retrospective 5-year study. J Adv Nurs 20: 462-468.

48. Van Heeringen C, Jannes S, Buylaert W, Henderick H, De Bacquer D, et al. (1995) The management of non-compliance with referral to out-patient after-care among attempted suicide patients: A controlled intervention study. Psychological Medicine 25: 963-970.

49. Arensman E, Kerkhof AJ, Hengeveld MW, Mulder JD (1995) Medically treated suicide attempts: a four year monitoring study of the epidemiology in The Netherlands. J Epidemiol Community Health 49: 285-289.

50. Nordstrom P, Samuelsson M, Asberg M (1995) Survival analysis of suicide risk after attempted suicide. Acta Psychiatrica Scandinavica 91: 336-340.

51. De Moore GM, Robertson AR (1996) Suicide in the 18 years after deliberate self-harm a prospective study. Br J Psychiatry 169: 489-494.

52. Johnsson Fridell E, Ojehagen A, Traskman-Bendz L (1996) A 5-year follow-up study of suicide attempts. Acta Psychiatr Scand 93: 151-157.

53. Hjelmeland H (1996) Repetition of parasuicide: a predictive study. Suicide & Life-Threatening Behavior 26: 395-404.

54. Palsson PS, Jonsdottir G, Petursson H (1996) The mortality risk of psychiatric emergency patients: A follow-up study. Nord J Psychiatry 50: 207-216.

55. Schmidtke A, Bille-Brahe U, DeLeo D, Kerkhof A, Bjerke T, et al. (1996) Attempted suicide in Europe: Rates, trends and sociodemographic characteristics of suicide attempters during the period 1989-1992. Results of the WHO/EURO Multicentre Study on Parasuicide. Acta Psychiatrica Scandinavica 93: 327-338.

56. Van der Sande R, Va Rooijen L, Buskens E, Allart E, Hawton K, et al. (1997) Intensive in-patient and community intervention versus routine care after attempted suicide. A randomised controlled intervention study. British Journal of Psychiatry 170: 35-41.

57. McEvedy C (1997) Trends in self-poisoning: Admissions to a central London hospital, 1991-1994. J R Soc Med 90: 496-498.

58. Gilbody S, House A, Owens D (1997) The early repetition of deliberate self harm. J R Coll Physicians Lond 31: 171-172.

59. Hawton K, Fagg J, Simkin S, Bale E, Bond A (1997) Trends in deliberate self-harm in Oxford, 1985-1995. Implications for clinical services and the prevention of suicide. Br J Psychiatry 171: 556-560.

60. Hjelmeland H, Stiles T, Bille-Brahe U, Ostamo A, Renberg E, et al. (1998) Parasuicide: The value of suicidal intent and various motives as predictors of future suicidal behaviour. Archives of Suicide Research 4: 209-225.

61. Batt A, Eudier F, Le Vaou P, Breurec J, Y., et al. (1998) Repetition of parasuicide: Risk factors in general hospital referred patients. Journal of Mental Health 7: 285-297.

62. Hall DJ, O'Brien F, Stark C, Pelosi A, Smith H (1998) Thirteen-year follow-up of deliberate self-harm, using linked data. Br J Psychiatry 172: 239-242.

63. Crawford MJ, Wessely S (1998) Does initial management affect the rate of repetition of deliberate self harm? cohort study. BMJ 317: 985.

64. Tejedor M, Diaz A, Castillon J, Pericay J (1999) Attempted suicide: Repetition and survival-findings of a follow-up study. Acta Psychiatrica Scandinavica 100: 205-211.

65. Salander Renberg E (1999) Parasuicide in a northern Swedish county 1989-1995 and its relation to suicide. Archives of Suicide Research 5: 97-112.

66. Curran S, Fitzgerald M, Greene V (1999) Psychopathology 8 1/2 years post parasuicide. Crisis 20: 115-120.

67. Kennedy P, Rogers B, Speer S, Frankel H (1999) Spinal cord injuries and attempted suicide: A retrospective review. Spinal Cord 37: 847-852.

68. Ruchholtz S, Pajonk FG, Waydhas C, Lewan U, Nast-Kolb D, et al. (1999) Long-term results and quality of life after parasuicidal multiple blunt trauma. Crit Care Med 27: 522-530.

69. Cusick T, Chang F, Woodson T, Helmer S (1999) Is resuscitation after traumatic suicide attempt a futile effort? A five-year review at a Level I trauma center. American Surgeon 65: 643-647.

70. Carter GL, Whyte IM, Ball K, Carter NT, Dawson AH, et al. (1999) Repetition of deliberate self-poisoning in an Australian hospital-treated population. Medical Journal of Australia 170: 307-311.

71. Gonzalez RB, Feal PG, Junquera FB, Santos MC, Iglesias JMS, et al. (2000) Suicides and suicides tentatives in Galicia as a result of pharmacs ingestion. Rev Psiquiatria Fac Med Barna 27: 27-25.

72. Dieserud G, Loeb M, Ekeberg O (2000) Suicidal behavior in the municipality of Baerum, Norway: a 12-year prospective study of parasuicide and suicide. Suicide Life Threat Behav 30: 61-73.

73. Evans J, Reeves B, Platt H, Leibenau A, Goldman D, et al. (2000) Impulsiveness, serotonin genes and repetition of deliberate self-harm (DSH). Psychol Med 30: 1327-1334.

74. Archinard M, Haynal-Reymond V, Heller M (2000) Doctor's and patients' facial expressions and suicide reattempt risk assessment. J Psychiatr Res 34: 261-262.

75. Morgan V, Coleman M (2000) An evaluation of the implementation of a liaison service in an A&E department. J Psychiatr Ment Health Nurs 7: 391-397.

76. Scocco P, Marietta P, Tonietto M, Dello Buono M, De Leo D (2000) The role of psychopathology and suicidal intention in predicting suicide risk: A longitudinal study. Psychopathology 33: 143-150.

77. Engeland A, Wiik J, Lande G (2001) Registration of parasuicide at hospitals and emergency units. Tidsskrift for den Norske laegeforening 121: 1033-1037.

78. Suokas J, Suominen K, Isometsa E, Ostamo A, Lonnqvist J (2001) Long-term risk factors for suicide mortality after attempted suicide--findings of a 14-year follow-up study. Acta Psychiatr Scand 104: 117-121.

79. AbuMadini MS, Rahim SJA (2001) Deliberate self-harm in a Saudi university Hospital: a case series over six years (1994-2000). The Arab Journal of Psychiatry 12: 22-35.

80. Guthrie E, Kapur N, Mackway-Jones K, Chew-Graham C, Moorey J, et al. (2001) Randomised controlled trial of brief psychological intervention after deliberate self poisoning. BMJ 323: 135-138.

81. Ostamo A, Lonnqvist J (2001) Excess mortality of suicide attempters. Social Psychiatry and Psychiatric Epidemiology 36: 29-35.

82. Carter GL, Clover KA, Bryant JL, Whyte IM (2002) Can the Edinburgh Risk of Repetition Scale predict repetition of deliberate self-poisoning in an Australian clinical setting? Suicide & Life-Threatening Behavior 32: 230-239.

83. Nimeus A, En M, Traskman-Bendz L (2002) High Suicidal Intent Scores Indicate Future Suicide. Archives of Suicide Research 6: 211-219.

84. Bennewith O, Stocks N, Gunnell D, Peters TJ, Evans MO, et al. (2002) General practice based intervention to prevent repeat episodes of deliberate self harm: cluster randomised controlled trial. BMJ 324: 1254-1257.

85. Antretter E, Dunkel D, Seibl R, Haring C (2002) The discriminant and predictive quality of suicide intent. A cluster-analytic approach. Nervenarzt 73: 219-230.

86. Liu L, Xiao S (2002) A follow-up study of suicide attemptors. Chinese Mental Health Journal 16: 253-256.

87. Jenkins G, Hale R, Papanastassiou M, Crawford M, Tyrer P (2002) Suicide rate 22 years after parasuicide: Cohort study. British Medical Journal 325: 1155.

88. Clarke T, Baker P, Watts C, Williams K, Feldman R, et al. (2002) Self-harm in adults: A randomised controlled trial of nurse-led case management versus routine care only. Journal of Mental Health 11: 167-176.

89. Hawton K, Harriss L, Hall S, Simkin S, Bale E, et al. (2003) Deliberate self-harm in Oxford, 1990-2000: a time of change in patient characteristics. Psychol Med 33: 987-995.

90. Kapur N, House A, May C, Creed F (2003) Service provision and outcome for deliberate self-poisoning in adults--results from a six centre descriptive study. Soc Psychiatry Psychiatr Epidemiol 38: 390-395.

91. Hawton K, Zahl D, Weatherall R (2003) Suicide following deliberate self-harm: long-term follow-up of patients who presented to a general hospital. Br J Psychiatry 182: 537-542.

92. Moron DJ (2003) Study of the suicide attempts treated in a sanatory area during a 9 year period. Anales de Psiquiatria 19: 431-439.

93. Keeley HS, O'Sullivan M, Corcoran P (2003) Background stressors and deliberate self-harm: Prospective case note study in southern Ireland. The Psychiatrist 27.

94. Tyrer P, Thompson S, Schmidt U, Jones V, Knapp M, et al. (2003) Randomized controlled trial of brief cognitive behaviour therapy versus treatment as usual in recurrent deliberate self-harm: The POPMACT study. Psychological Medicine 33: 969-976.

95. Horrocks J, Price S, House A, Owens D (2003) Self-injury attendances in the accident and emergency department: Clinical database study. British Journal of Psychiatry 183: 34-39.

96. Monti K, Cedereke M, Ojehagen A (2003) Treatment Attendance and Suicidal Behavior 1 Month and 3 Months After a Suicide Attempt: A Comparison Between Two Samples. Archives of Suicide Research 7: 167-174.

97. Dieserud G, Roysamb E, Braverman MT, Dalgard OS, Ekeberg O (2003) Predicting Repetition of Suicide Attempt: A Prospective Study of 50 Suicide Attempters. Archives of Suicide Research 7: 1-15.

98. Suominen K, Isometsa E, Haukka J, Lonnqvist J (2004) Substance use and male gender as risk factors for deaths and suicide--a 5-year follow-up study after deliberate self-harm. Soc Psychiatry Psychiatr Epidemiol 39: 720-724.

99. Courtet P, Picot M, C., Bellivier F, Torres S, et al. (2004) Serotonin transporter gene may be involved in short-term risk of subsequent suicide attempts. Biol Psychiatry 55: 46-51.

100. Henriques GR, Brown GK, Berk MS, Beck AT (2004) Marked increases in psychopathology found in a 30-year cohort comparison of suicide attempters. Psychological Medicine 34: 833-841.

101. Corcoran P, Keeley HS, O'Sullivan M, Perry IJ (2004) The incidence and repetition of attempted suicide in Ireland. European Journal of Public Health 14: 19-23.

102. Soderberg S, Kullgren G, Salander Renberg E (2004) Childhood sexual abuse predicts poor outcome seven years after parasuicide. Social Psychiatry and Psychiatric Epidemiology 39: 916-920.

103. Ito T, Hada M, Kimura A, Kurosawa H, Okubo Y (2004) The study of suicide attempters in a critical care medical center and their prognosis. Seishin Igaku 46: 389-396.

104. Beautrais AL (2004) Further suicidal behavior among medically serious suicide attempters. Suicide & Life-Threatening Behavior 34: 1-11.

105. Suominen K, Isometsa E, Ostamo A, Lonnqvist J (2004) Level of suicidal intent predicts overall mortality and suicide after attempted suicide: A 12-year follow-up study. BMC Psychiatry 4.

106. Reith DM, Whyte I, Carter G, McPherson M, Carter N (2004) Risk factors for suicide and other deaths following hospital treated self-poisoning in Australia. Aust N Z J Psychiatry 38: 520-525.

107. Suominen K, Isometsa E, Suokas J, Haukka J, Achte K, et al. (2004) Completed Suicide after a Suicide Attempt: A 37-Year Follow-Up Study. American Journal of Psychiatry 161: 562-563.

108. Skogman K, Alsen M, Ojehagen A (2004) Sex differences in risk factors for suicide after attempted suicide-a follow-up study of 1052 suicide attempters. Social Psychiatry and Psychiatric Epidemiology 39: 113-120.

109. Brown GK, Ten Have T, Henriques GR, Xie SX, Hollander JE, et al. (2005) Cognitive therapy for the prevention of suicide attempts: a randomized controlled trial. JAMA 294: 563-570.

110. Evans J, Evans M, Morgan H, Hayward A, Gunnell D (2005) Crisis card following self-harm: 12-Month follow-up of a randomised controlled trial. British Journal of Psychiatry 187: 186-187.

111. Carter GL, Clover K, Whyte IM, Dawson AH, D'Este C (2005) Postcards from the EDge project: randomised controlled trial of an intervention using postcards to reduce repetition of hospital treated deliberate self poisoning. BMJ 331: 805.

112. Gibb SJ, Beautrais AL, Fergusson DM (2005) Mortality and further suicidal behaviour after an index suicide attempt: a 10-year study. Australian & New Zealand Journal of Psychiatry 39: 95-100.

113. Harriss L, Hawton K (2005) Suicidal intent in deliberate self-harm and the risk of suicide: the predictive power of the Suicide Intent Scale. J Affect Disord 86: 225-233.

114. Leslie S, Greig L, Mackie R, Gotz M, Morrison D (2005) A survey of admissions following self-poisoning. Psychiatric Bulletin 29: 305-308.

115. Owens D, Wood C, Greenwood D, Hughes T, Dennis M (2005) Mortality and suicide after non-fatal self-poisoning: 16-Year outcome study. British Journal of Psychiatry 187: 470-475.

116. Kapur N, Cooper J, King-Hele S, Webb R, Lawlor M, et al. (2006) The repetition of suicidal behavior: a multicenter cohort study. J Clin Psychiatry 67: 1599-1609.

117. Benjaminsen S, Knudsen A, Thomsen R, Balslov K (2006) Prevention of repeated suicide attempts. Evaluation of the treatment effectiveness. Ugeskrift for laeger 168: 553-558.

118. Wang A, Mortensen G (2006) Core features of repeated suicidal behaviour: A long term follow-up after suicide attempts in a low-suicide-incidence population. Social Psychiatry and Psychiatric Epidemiology 41: 103-107.

119. Tiihonen J, Lonnqvist J, Wahlbeck K, Klaukka T, Tanskanen A, et al. (2006) Antidepressants and the risk of suicide, attempted suicide, and overall mortality in a nationwide cohort. Archives of General Psychiatry 63: 1358-1367.

120. Johnston A, Cooper J, Webb R, Kapur N (2006) Individual- and area-level predictors of self-harm repetition. Br J Psychiatry 189: 416-421.

121. Pulido FR, Abad MEM, de Chaves Gonzalez FG, Hernandez DM, Davila EG (2006) The epidemiology of parasuicide in Canary Islands. The European Journal of Psychiatry.20: pp.

122. Vaiva G, Ducrocq F, Meyer P, Mathieu D, Philippe A, et al. (2006) Effect of telephone contact on further suicide attempts in patients discharged from an emergency department: Randomised controlled study. British Medical Journal 332: 1241-1244.

123. Belgamwar RB, Hodgson RE, Waters K (2006) Trends and characteristics of deliberate self-harm hospital presentations in an English County. International Journal of Psychiatry in Clinical Practice.10: pp.

124. Eudier F, Gault S, Batt-Moillo A, Drapier D, Millet B (2006) Reduction in short-term repetition of attempted suicide associated with new organization of psychiatric management of suicide attempters: Two cohorts. Presse Medicale 35: 759-763.

125. Carter GL, Clover K, Whyte IM, Dawson AH, D'Este C (2007) Postcards from the EDge: 24-month outcomes of a randomised controlled trial for hospital-treated self-poisoning. Br J Psychiatry 191: 548-553.

126. Loas G (2007) Anhedonia and suicide: A 6.5-Yr. Follow-up study of patients hospitalised for a suicide attempt. Psychological Reports 100: 183-190.

127. Haw C, Bergen H, Casey D, Hawton K (2007) Repetition of deliberate self-harm: a study of the characteristics and subsequent deaths in patients presenting to a general hospital according to extent of repetition. Suicide Life Threat Behav 37: 379-396.

128. Christiansen E, Jensen BF (2007) Risk of repetition of suicide attempt, suicide or all deaths after an episode of attempted suicide: a register-based survival analysis. Aust N Z J Psychiatry 41: 257-265.

129. Lindqvist D, Nimeus A, Traskman-Bendz L (2007) Suicidal intent and psychiatric symptoms among inpatient suicide attempters. Nordic Journal of Psychiatry 61: 27-32.

130. Caldera T, Herrera A, Kullgren G, Renberg ES (2007) Suicide intent among parasuicide patients in Nicaragua: a surveillance and follow-up study. Archives of Suicide Research 11: 351-360.

131. Haukka J, Suominen K, Partonen T, Lonnqvist J (2008) Determinants and outcomes of serious attempted suicide: a nationwide study in Finland, 1996-2003. Am J Epidemiol 167: 1155-1163.

132. Howson M, Yates K, Hatcher S (2008) Re-presentation and suicide rates in emergency department patients who self-harm. EMA - Emergency Medicine Australasia 20: 322-327.

133. Chandrasekaran R, Gnanaselane J (2008) Predictors of repeat suicidal attempts after first-ever attempt: A two-year follow-up study. Hong Kong Journal of Psychiatry.18: pp.

134. McAuliffe C, Corcoran P, Hickey P, McLeavey BC (2008) Optional thinking ability among hospital-treated deliberate self-harm patients: a 1-year follow-up study. Br J Clin Psychol 47: 43-58.

135. Payne RA, Oliver JJ, Bain M, Elders A, Bateman DN (2009) Patterns and predictors of re-admission to hospital with self-poisoning in Scotland. Public Health 123: 134-137.

136. Hvid M, Wang A (2009) Preventing repetition of attempted suicide--I. Feasibility (acceptability, adherence, and effectiveness) of a Baerum-model like aftercare. Nordic Journal of Psychiatry 63: 148-153.

137. Heyerdahl F, Bjornaas MA, Dahl R, Hovda KE, Nore AK, et al. (2009) Repetition of acute poisoning in Oslo: 1-year prospective study. Br J Psychiatry 194: 73-79.

138. Nakagawa M, Yamada T, Yamada S, Natori M, Hirayasu Y, et al. (2009) Follow-up study of suicide attempters who were given crisis intervention during hospital stay: Pilot study. Psychiatry and Clinical Neurosciences 63: 122-123.

139. Antretter E, Dunkel D, Haring C (2009) Cause-specific excess mortality in suicidal patients: gender differences in mortality patterns. Gen Hosp Psychiatry 31: 67-74.

140. Prescott K, Stratton R, Freyer A, Hall I, Le Jeune I (2009) Detailed analyses of self-poisoning episodes presenting to a large regional teaching hospital in the UK. Br J Clin Pharmacol 68: 260-268.

141. Scoliers G, Portzky G, van Heeringen K, Audenaert K (2009) Sociodemographic and psychopathological risk factors for repetition of attempted suicide: a 5-year follow-up study. Archives of suicide research : official journal of the International Academy for Suicide Research 13: 201-213.

142. Bjornaas MA, Jacobsen D, Haldorsen T, Ekeberg O (2009) Mortality and causes of death after hospital-treated self-poisoning in Oslo: a 20-year follow-up. Clin Toxicol (Phila) 47: 116-123.

143. Bergen H, Hawton K, Waters K, Cooper J, Kapur N (2010) Epidemiology and trends in non-fatal self-harm in three centres in England: 2000-2007. Br J Psychiatry 197: 493-498.

144. Waern M, Sjostrom N, Marlow T, Hetta J (2010) Does the Suicide Assessment Scale predict risk of repetition? A prospective study of suicide attempters at a hospital emergency department. European Psychiatry 25: 421-426.

145. Crawford MJ, Csipke E, Brown A, Reid S, Nilsen K, et al. (2010) The effect of referral for brief intervention for alcohol misuse on repetition of deliberate self-harm: an exploratory randomized controlled trial. Psychol Med 40: 1821-1828.

146. Mehlum L, Jorgensen T, Diep L, Nrugham L (2010) Is organizational change associated with increased rates of readmission to general hospital in suicide attempters? a 10-year prospective catchment area study. Archives of Suicide Research 14: 171-181.

147. Bergen H, Hawton K, Waters K, Cooper J, Kapur N (2010) Psychosocial assessment and repetition of self-harm: the significance of single and multiple repeat episode analyses. J Affect Disord 127: 257-265.

148. Onen Sertoz O, Noyan M, Sertoz N, Elbi H (2010) Can suicide attempts be predicted? The results of the six-month prospective follow-up of patients who had attempted suicide and admitted to emergency service of a university hospital. Anadolu Psikiyatri Dergisi 11: 1-8.

149. Chen VC, Tan HK, Cheng AT, Chen CY, Liao LR, et al. (2010) Non-fatal repetition of self-harm: population-based prospective cohort study in Taiwan. British Journal of Psychiatry 196: 31-35.

150. Beautrais AL, Gibb SJ, Faulkner A, Fergusson DM, Mulder RT (2010) Postcard intervention for repeat self-harm: randomised controlled trial. Br J Psychiatry 197: 55-60.

151. Russell G, Owens D (2010) Psychosocial assessment following self-harm: repetition of nonfatal self-harm after assessment by psychiatrists or mental health nurses. Crisis 31: 211-216.

152. Sverrisson KO, Palsson SP, Sigvaldason K, Karason S (2010) [Clinical aspects and follow up of suicide attempts treated in a general intensive care unit at Landspitali University Hospital in Iceland 2000-2004]. Laeknabladid 96: 101-107.

153. Bertolote JM, Fleischmann A, De Leo D, Phillips MR, Botega NJ, et al. (2010) Repetition of suicide attempts: Data from emergency care settings in five culturally different low- and middle-income countries participating in the WHO SUPRE-MISS study. Crisis: The Journal of Crisis Intervention and Suicide Prevention.31: pp.

154. Runeson B, Tidemalm D, Dahlin M, Lichtenstein P, Langstrom N (2010) Method of attempted suicide as predictor of subsequent successful suicide: national long term cohort study. BMJ 341: c3222.

155. Johannessen HA, Dieserud G, De Leo D, Claussen B, Zahl PH (2011) Chain of care for patients who have attempted suicide: a follow-up study from Baerum, Norway. BMC Public Health 11: 81.

156. Hatcher S, Sharon C, Parag V, Collins N (2011) Problem-solving therapy for people who present to hospital with self-harm: Zelen randomised controlled trial. Br J Psychiatry 199: 310-316.

157. Lee Y, Lin P, Y., Yeh W, C., et al. (2012) Repeated suicide attempts among suicidal cases: Outcome of one-year follow-up. Asia-Pacific Psychiatry 4: 174-180.

158. Karasouli E, Owens D, Abbott RL, Hurst KM, Dennis M (2011) All-cause mortality after non-fatal self-poisoning: a cohort study. Soc Psychiatry Psychiatr Epidemiol 46: 455-462.

159. Hvid M, Vangborg K, Sorensen H, Nielsen I, Stenborg J, et al. (2011) Preventing repetition of attempted suicideII. the Amager Project, a randomized controlled trial. Nordic Journal of Psychiatry 65: 292-298.

160. Maier R, Stieglitz RD, Marsch SC, Rlecher-Rossier A (2011) Patients in intensive care after a suicide attempt with legal drugs - risk profile and course. Fortschritte der neurologie, psychiatrie 79: 283-298.

161. Yip PS, Hawton K, Liu K, Liu KS, Ng PW, et al. (2011) A study of deliberate self-harm and its repetition among patients presenting to an emergency department. Crisis 32: 217-224.

162. Hassanian-Moghaddam H, Sarjami S, Kolahi AA, Carter GL (2011) Postcards in Persia: randomised controlled trial to reduce suicidal behaviours 12 months after hospital-treated self-poisoning. Br J Psychiatry 198: 309-316.

163. Chung CH, Lai CH, Chu CM, Pai L, Kao S, et al. (2012) A nationwide, population-based, long-term follow-up study of repeated self-harm in Taiwan. BMC Public Health 12: 744.

164. Kuo C, J., Gunnell D, Chen C, C., et al. (2012) Suicide and non-suicide mortality after self-harm in Taipei City, Taiwan. British Journal of Psychiatry 200: 405-411.

165. Cebria AI, Parra I, Pamias M, Escayola A, Garcia-Pares G, et al. (2013) Effectiveness of a telephone management programme for patients discharged from an emergency department after a suicide attempt: controlled study in a Spanish population. J Affect Disord 147: 269-276.

166. Monnin J, Thiemard E, Vandel P, Nicolier M, Tio G, et al. (2012) Sociodemographic and psychopathological risk factors in repeated suicide attempts: gender differences in a prospective study. J Affect Disord 136: 35-43.

167. Sjostrom N, Hetta J, Waern M (2012) Sense of coherence and suicidality in suicide attempters: A prospective study. Journal of Psychiatric and Mental Health Nursing 19: 62-69.

168. Perry IJ, Corcoran P, Fitzgerald AP, Keeley HS, Reulbach U, et al. (2012) The incidence and repetition of hospital-treated deliberate self harm: findings from the world's first national registry. PLoS One 7: e31663.

169. Stefansson J, Nordstrom P, Jokinen J (2012) Suicide Intent Scale in the prediction of suicide. Journal of Affective Disorders 136: 167-171.

170. Bilen K, Ponzer S, Ottosson C, Castren M, Pettersson H (2013) Deliberate self-harm patients in the emergency department: who will repeat and who will not? Validation and development of clinical decision rules. Emerg Med J 30: 650-656.

171. Morthorst B, Krogh J, Erlangsen A, Alberdi F, Nordentoft M (2012) Effect of assertive outreach after suicide attempt in the AID (assertive intervention for deliberate self harm) trial: randomised controlled trial. BMJ 345: e4972.

172. Isung J, Mobarrez F, Nordstrom P, Asberg M, Jokinen J (2012) Low plasma vascular endothelial growth factor (VEGF) associated with completed suicide. World J Biol Psychiatry 13: 468-473.

173. Riedi G, Mathur A, Seguin M, Bousquet B, Czapla P, et al. (2012) Alcohol and repeated deliberate self-harm: preliminary results of the French cohort study of risk for repeated incomplete suicides. Crisis 33: 358-363.

174. Pan YJ, Chang WH, Lee MB, Chen CH, Liao SC, et al. (2012) Effectiveness of a nationwide aftercare program for suicide attempters. Psychol Med: 1-8.

175. Bergen H, Hawton K, Kapur N, Cooper J, Steeg S, et al. (2012) Shared characteristics of suicides and other unnatural deaths following non-fatal self-harm? A multicentre study of risk factors. Psychol Med 42: 727-741.

176. Choi JW, Park S, Yi KK, Hong JP (2012) Suicide mortality of suicide attempt patients discharged from emergency room, nonsuicidal psychiatric patients discharged from emergency room, admitted suicide attempt patients, and admitted nonsuicidal psychiatric patients. Suicide & Life-Threatening Behavior 42: 235-243.

177. Jimenez-Trevino L, Saiz P, Corcoran P, Garcia-Portilla M, Buron P, et al. (2012) The incidence of hospital-treated attempted suicide in Oviedo, Spain. Crisis 33: 46-53.
